# 3D characterisation and quantification of an offshore freshened groundwater system in the Canterbury Bight

**DOI:** 10.1038/s41467-020-14770-7

**Published:** 2020-03-13

**Authors:** Aaron Micallef, Mark Person, Amir Haroon, Bradley A. Weymer, Marion Jegen, Katrin Schwalenberg, Zahra Faghih, Shuangmin Duan, Denis Cohen, Joshu J. Mountjoy, Susanne Woelz, Carl W. Gable, Tanita Averes, Ashwani Kumar Tiwari

**Affiliations:** 1 Marine Geology & Seafloor Surveying, Department of Geosciences, University of Malta, Msida, Malta; 2 Helmholtz Centre for Ocean Research, GEOMAR, Kiel, Germany; 3 Hydrology Program, New Mexico Tech, Socorro, NM USA; 4 Federal Institute for Geosciences and Natural Resources (BGR), Hanover, Germany; 5 National Institute for Water and Atmospheric Research (NIWA), Wellington, New Zealand; 6 Computational Earth Science, Earth and Environmental Sciences Division, Los Alamos National Laboratory, Los Alamos, NM USA; 7 Institute of Geosciences, Christian-Albrechts-Universität zu Kiel, Kiel, Germany; 8 Department of Environment, Land and Infrastructure Engineering, Politecnico di Torino, Turin, Italy

**Keywords:** Hydrology, Ocean sciences, Solid Earth sciences

## Abstract

Although offshore freshened groundwater (OFG) systems have been documented in numerous continental margins worldwide, their geometry, controls and emplacement dynamics remain poorly constrained. Here we integrate controlled-source electromagnetic, seismic reflection and borehole data with hydrological modelling to quantitatively characterise a previously unknown OFG system near Canterbury, New Zealand. The OFG system consists of one main, and two smaller, low salinity groundwater bodies. The main body extends up to 60 km from the coast and a seawater depth of 110 m. We attribute along-shelf variability in salinity to permeability heterogeneity due to permeable conduits and normal faults, and to recharge from rivers during sea level lowstands. A meteoric origin of the OFG and active groundwater migration from onshore are inferred. However, modelling results suggest that the majority of the OFG was emplaced via topographically-driven flow during sea level lowstands in the last 300 ka. Global volumetric estimates of OFG will be significantly revised if active margins, with steep coastal topographies like the Canterbury margin, are considered.

## Abstract (web summary)

The authors here combine a range of geophysical data, numerical modelling and borehole data to present a high resolution map of an offshore freshened groundwater system in the Canterbury Bight, New Zealand. The study shows the extensions of the offshore freshened groundwater system to be controlled by high permeability shelf sediments, buried paleochannels and onshore rivers.

## Introduction

Vast offshore bodies of fresh and moderately brackish groundwater (concentration of total dissolved solids of <10 g l^−1^) have been documented up to 100 km from modern shorelines and down to 4.5 km below the seafloor (bsf)^1^. The majority of offshore freshened groundwater (OFG) is hosted in shallow (<300 m), poorly consolidated, clastic sediments in seawater depths less than 50 m^1–3^. Nearly all discoveries of OFG have been made along passive continental margins, mostly in the Atlantic US and European margins^4,5^. There are at least five mechanisms known to be responsible for the emplacement of OFG. These include active groundwater migration across topographic gradients via present day, permeable connections between offshore and onshore aquifers^6^, recharge during Pleistocene sea-level lowstands^7,8^, sub-glacial and pro-glacial injection^9^, entrapment of connate water in subsiding basins^10^, and gas hydrate dissociation^11^. Global volumetric estimates of OFG were derived in passive margins and are on the order of 10^5^ km^3^. This is two orders of magnitude greater than the volume of groundwater that has been extracted globally from continental aquifers since 1900 (refs. ^1,8^).

The main driving force for an improved understanding of OFG systems is their potential use as a source of potable water^12^. Groundwater resources are declining in terms of quantity and quality as a result of climate change, pollution and over-exploitation caused by population growth and urbanisation, particularly in coastal regions and island nations^13,14^. OFG may provide a buffer to increased demand during periods of intense drought and, in some coastal areas, is already being inadvertently exploited by onshore pumping^3,15^. Industrial sectors involving seafloor engineering, carbon dioxide sequestration, and ore deposit and petroleum exploration have a direct interest in the evolution of OFG systems because these can place better constraints on past fluid migration histories^16^. Apart from these broad societal impacts, OFG plays a fundamental role in biogeochemical fluxes to the ocean as well as benthic and sub-seafloor ecology^17^. OFG can also provide potential archives of former environmental conditions^18^ and contribute to advance our understanding of human settlement and migration in the past^19^.

The characteristics and dynamics of OFG systems remain poorly constrained. There are many first-order questions waiting to be addressed, mainly related to the geometry, distribution and extent of offshore aquifers, as well as their flow and emplacement dynamics. This stems from the fact that our understanding of OFG systems is predominantly based on offshore borehole data from legacy drilling campaigns and incidental discoveries in petroleum wells^20^. The coverage of these borehole data is limited^1^, and direct observation of the aquifer structure and geochemical characteristics of OFG remain rare^21,22^. In addition, most measurements and research efforts related to OFG have focused on the nearshore zone^23^. The control of the geological environment on the spatial distribution and flow of OFG is also poorly constrained^22,24^. Contrasting results have been reported, with freshwater being preferentially stored in coarse-grained sandy deposits^20^ or in fine-grained clay intervals^22^, and with faults acting as both barriers and conduits^25^.

Controlled source electromagnetic methods using a horizontal electric dipole source are sensitive to bulk electrical resistivity and can detect resistivity contrasts between the OFG and the surrounding seawater-saturated sediment, in a similar manner to hydrocarbon reservoirs^26,27^. In this study we integrate offshore time-domain controlled-source electromagnetic (CSEM) data with multichannel seismic reflection data, borehole data and hydrological modelling to quantitatively characterise an OFG system at high spatial resolution. The objectives of our study are to constrain the 3D geometry, extent, dimensions, hydraulic and age characteristics of the OFG system, to infer the origin and emplacement mechanisms of the OFG, and to identify the controls of the OFG system and its characteristics. Our study area is the Canterbury Bight, located off the eastern coast of the South Island of New Zealand. This continental margin was investigated because a pore water salinity anomaly was recorded in borehole U1353 during IODP expedition 317 (refs. ^1,28^). Here we document an extensive OFG system that consists of one main, and two smaller, low salinity groundwater bodies. The origin of the OFG is meteoric, although the majority of the OFG appears to have been emplaced via topographically driven flow during sea-level lowstands in the last 300 ka. We also report along-shelf variability in OFG salinity, which we attribute to permeability heterogeneity due to permeable conduits and normal faults, and to recharge from rivers during sea-level lowstands.

## Results

### Geological framework

**Fig. 1:**
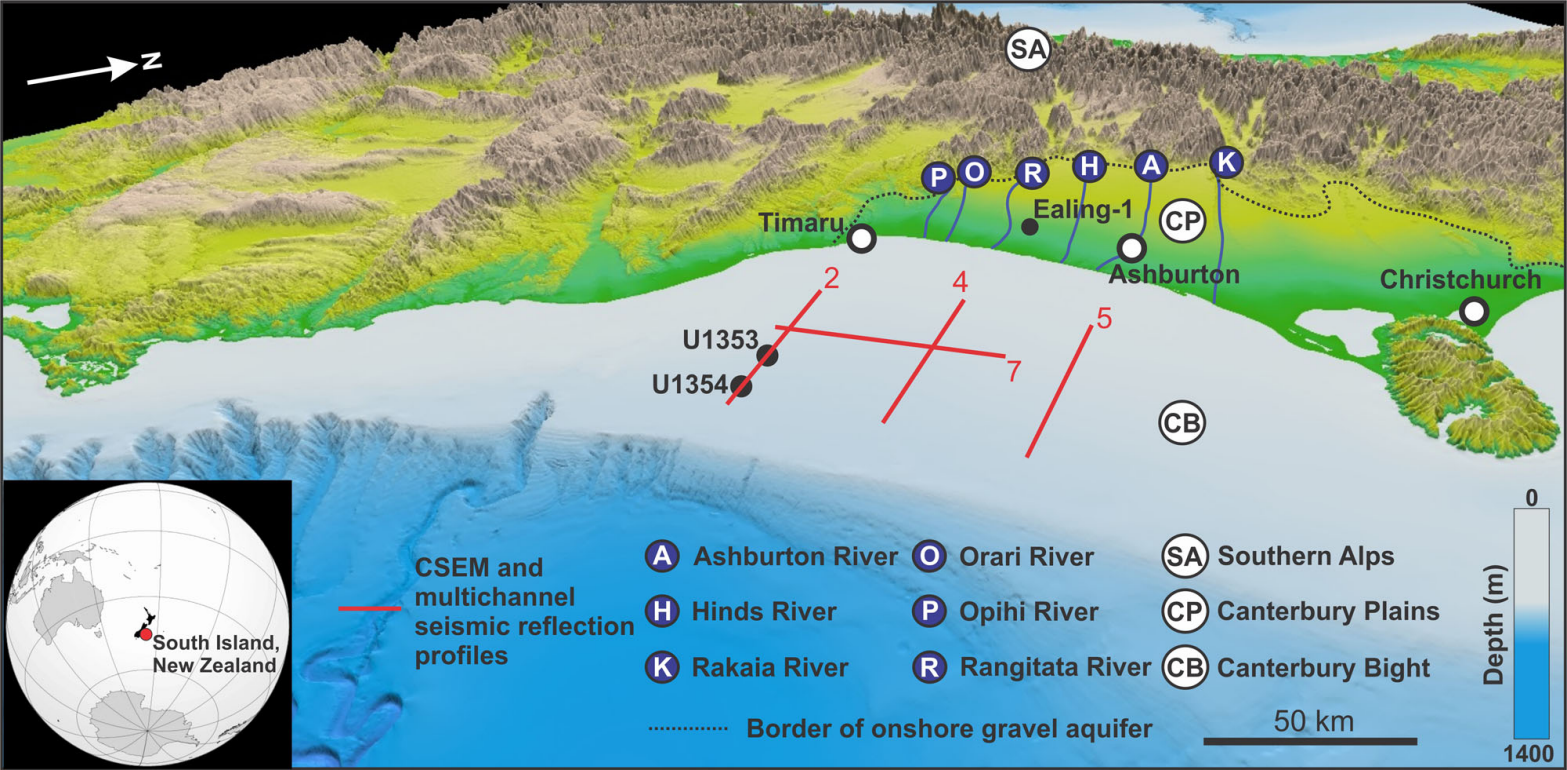
Study area. Three-dimensional digital elevation model of the Canterbury Basin (Source: https://data/linz.govt.nz). The location of the rivers, onshore gravel aquifer, onshore well Ealing-1, CSEM and multichannel seismic reflection lines, and boreholes U1353 and U1354, is shown.

The 50,000 km^2^ Canterbury Basin is a foreland basin on the eastern side of the Southern Alps in the South Island of New Zealand (Fig. 1). The basin includes the Canterbury Plains onshore and the Canterbury Bight shelf and continental slope offshore^29,30^. The onshore sedimentary sequence is dominated by a >600-m-thick Quaternary, cyclically stacked, fluvio-deltaic succession, which includes an alternation of gravels and sand/silt units^31^. These sediments were eroded from Torlesse rocks in the >3500 m high Southern Alps during glacial times, transported by high energy braided rivers, and deposited as glacial outwash onto a margin subsiding at 0.2–0.5 m ka^−1^ (refs. ^30,32^). The main aquifers are hosted in gravels down to at least 150 m depth^33^, with unconnected sand and silt/clay layers forming aquitards^30,34,35^. The regional flow of groundwater in the Canterbury aquifers is from the foothills of the Southern Alps towards the sea^34^.

The Canterbury Bight comprises a continental shelf that is 180 km long and up to 95 km wide, with a shelf gradient of 0.09° and a maximum depth of 140–150 m^30^ (Fig. 1). It comprises a 1-km-thick progradational succession of coeval shelf-slope deposits that are punctuated by numerous advances of the braid plain (up to 70 km eastwards) during periods of sea-level fall^30^. In spite of its proximity to the Alpine Fault plate boundary, the Canterbury Bight has been an area of relative tectonic stability since the late Cretaceous^36^. The sedimentary history of the Canterbury Bight since the middle Miocene has been controlled by eustatically driven transgressive–regressive cycles^37–39^. The sedimentary sequence within this time frame can be divided into 19 regional sequence-bounding unconformities identified from seismic reflection profiles, which represent erosional surfaces caused by marine ravinement superposed on subaerial exposure surfaces^30,37^. The most recent 15 of these unconformities have been correlated with borehole logs from IODP expedition 317 and correspond to coarse sandy or shelly beds overlain by fining upwards mud and sandy mud^37–39^.

**Fig. 2:**
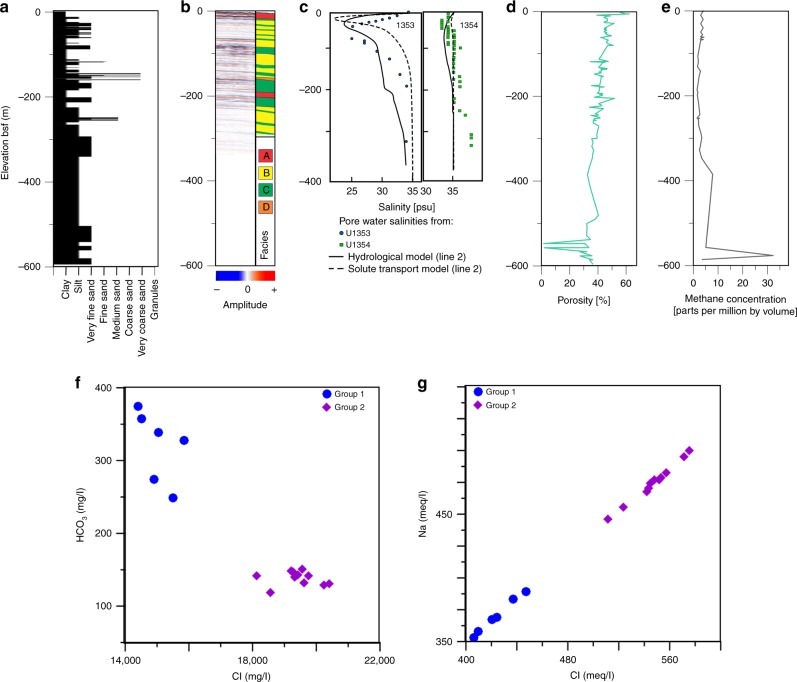
Borehole U1353 data. Depth profiles of **a** sediment grain size, **b** seismic reflection data (from TAN1703 survey) and interpreted facies, **c** pore water salinity, **d** porosity, **e** methane concentration, **f** plots of HCO_3_ vs. Cl and **g** Na vs. Cl. Pore water salinity profiles, derived from the hydrological model (solid line) and the model for solute transport by vertical diffusion (dashed line) for the site of boreholes U1353 and U1354, are included in **c**.

Boreholes U1353 and U1354 were drilled in the middle to outer Canterbury Bight during IODP expedition 317 (ref. ^40^; Fig. 1). Downhole variations in sediment grain size, pore water salinity, porosity and methane concentration for borehole U1353 are displayed in Fig. 2a–e. Variations in chemical element concentration for pore water samples from borehole U1353 are listed in Supplementary Table 1. The sediments were deposited in inner to outer shelf settings between the Holocene and Early Miocene, and predominantly consist of silt and clay with sub-ordinate layers of sand and granules^40^. Down to 180 m bsf, porosity values decrease from 45% to 40%. Pore water salinities decrease rapidly from 34 psu (practical salinity unit) at the seafloor to 24 psu at a depth of ~40 m bsf (psu, or practical salinity unit, is a unitless quantity equivalent to parts per thousand or g kg^−1^ and based on the properties of sea water conductivity; the global ocean has an average salinity of 35.5 psu). Salinity remains constant at 24–25 psu down to ~65 m bsf, and then increases gradually to 34 psu at ~180 m bsf. Downcore variations in salinity are mirrored by those in Cl^−^ and Na^+^ concentrations^40^. No pore water salinity anomaly was observed in borehole U1354 (Fig. 2c).

### Seismic reflection data

**Fig. 3:**
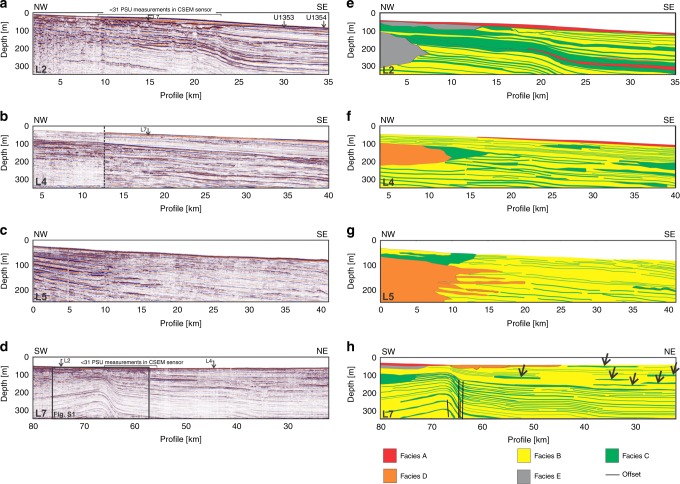
Multichannel seismic reflection profiles. **a**–**d** Processed seismic reflection profiles along lines 2, 4, 5 and 7. Location in Fig. 1. Location of intersecting profiles, boreholes U1353 and U1354, and low water column salinity values (derived from the conductivity–temperature–depth sensor on the CSEM instrument) are shown. The dotted line in L4 marks the boundary between two seismic reflection profiles acquired using different acquisition equipment and geometry, and in different weather conditions. **e**–**h** Interpreted facies. Offsets in seismic reflectors in line 7 are marked by black lines. Black arrows denote interpreted buried valleys infilled by coarse-grained sediments.

**Fig. 4:**
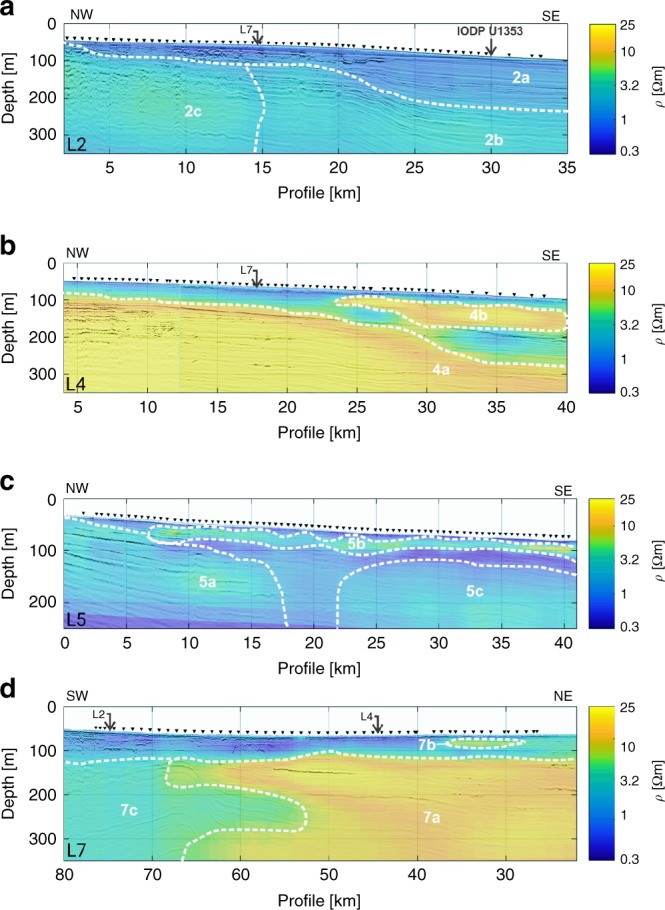
CSEM lines. Interpreted resistive features from the data acquired along lines 2, 4, 5, and 7, overlaid on seismic reflection profiles (see Table 1). The features are denoted by white labels whereas their boundaries are delineated by broken white lines. Location in Fig. 1. Location of intersecting lines and borehole U1353 is shown. Black triangles mark seafloor CSEM transmitter positions on waypoints.

Four seafloor transects across the Canterbury Bight (lines 2, 4, 5 and 7), with a total length of 175 km, were surveyed with multichannel reflection seismics and a seafloor-towed CSEM system (Figs. 1, 3a–d and 4). The multichannel seismic reflection profiles were sub-divided into five different facies (Fig. 3e–h; see Methods). Four of these facies were correlated with lithologies in boreholes U1353 and U1354: Facies A—clay; Facies B—silt; Facies C—fine sand; and Facies D—coarse sand/granules (Fig. 2b). Facies E was not sampled by the boreholes, but we interpret this as gravel. Our interpretation is based on the occurrence of a 600-m-thick alluvial gravel layer in the coastal well Ealing-1 (Fig. 1)^41^, which is expected to extend offshore, the similarity of facies E with the seismic signature of the 600-m-thick gravel layer onshore^42^, and the prediction of offshore gravel distribution from conceptual and quantitative stratigraphic models^30^. The stratigraphic framework across the Canterbury Bight therefore consists of an alternation between lowstand fluvial gravels and sands, which become thicker towards the shore, and highstand sands, silts and clays, which are more dominant in the deeper sections. This distribution of facies represents a trend of decreasing grain size and permeability with distance from the shoreline, and is consistent with sediment transport models results^30^. In line 7, we also observe 5–10 km long isolated bodies of facies C occurring at multiple depths and offset seismic reflectors at four locations in the SW section (Fig. 3h, Supplementary Fig. 1).

### CSEM data

We identify a number of resistive features in the CSEM inversion models, which we define as bodies of resistivity of >2 Ωm^43^ that extend across a minimum horizontal distance of 5 km (Fig. 4). The characteristics of these resistive features are summarised in Table 1. Line 4 displays the highest resistivity of the shore-normal profiles. Line 7 is characterised by shore-parallel variability in resistivity, with the highest values recorded in the vicinity of line 4. Resistive features correspond to all five seismic facies, with the most common being facies B and C (silt and fine sand). The thin, shallow, high resistivity features (4b and 7b) in lines 4 and 7 correspond to facies C (fine sand) (Figs. 3f, h and 4b, d). The top boundaries of the resistive features tend to follow seismic reflectors, although a few exceptions do occur (Fig. 4, Table 1).

**Table 1:** List of resistive features identified in lines 2, 4, 5 and 7 and their properties.

Line	Resistive feature	Resistivity of feature (Ωm)	Extent of feature (km)	Depth (bsf) of top of feature (m)	Thickness of feature (m)	Corresponding facies	Notes
2	2a	2	35	0	<50 in NW; 150 m in the SE	A, B, C; shallow and irregular u-shaped depressions infilled with facies E in the NW	
	2b	4	20	50–150	Up to 200	A, B, C, D	
	2c	8	15	50	>200	B, C, E	
4	4a	>20	37	25–150	>200	B, C, D	Top is parallel to seismic reflectors up to 35 km mark, then it deepens by 50 m
	4b	>20	15	25	50	B, C; NW section predominantly corresponds to facies C	
5	5a	3	17	0–100	>150	B, C, D; W section predominantly corresponds to facies D	
	5b	12	33	0–30	<25	B, C	Resistive feature has an irregular, wavy shape along its top and bottom
	5c	2	18	75	150	B, C	
7	7a	>20	40	~50	>200	B, C	SW boundary corresponds to 120 m vertical offset; top is parallel to seismic reflectors up to 65 km mark
	7b	20	10	~10	<25	B, C	
	7c	3	27	50–200	~50	B, C	Interfingers with 7a

Resistive features in the CSEM models can arise from low salinity pore water, a decrease in porosity (due to changes in grain size or presence of gas), and a decrease in clay content^44,45^. We do not find sufficient indicators that link the resistive features to the occurrence of gas-charged sediments. For lines 4 and 7, this would require high gas saturations of the available pore space in the order of 50%. There is only a low spatial correlation, in both the vertical and horizontal planes, between sub-seafloor indicators of gas in the seismic reflection data (e.g. acoustic blanking, amplitude anomalies, pipe structures) and the resistive features^46^. In addition, no significant amounts of hydrocarbons above background laboratory air were detected in the uppermost 350 m of borehole U1353 (ref. ^28^) (Fig. 2e).

**Fig. 5:**
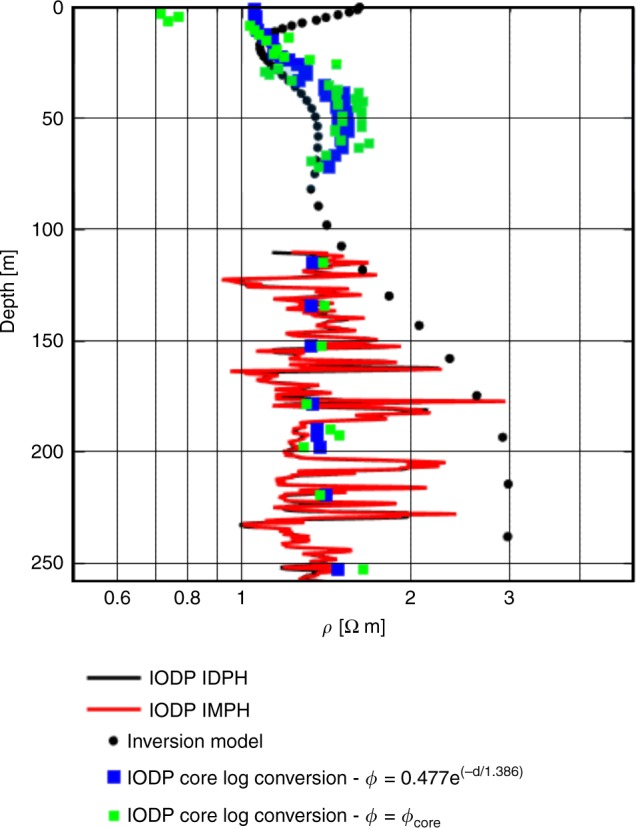
Validation of the resistivity-depth variations data for line 2 with resistivity measurements from borehole U1353. Downcore variations in resistivity derived from CSEM measurements (inversion model, shown as black circles), measured resistivities in borehole U1353 (IDPH = phasor deep induction log, shown as black line; IMPH = phasor medium induction log, shown as red line), and resistivities based on estimates using pore water salinity values from U1353 and the porosity–depth function derived from U1353, shown as blue squares, or the measured porosity values from U1353, shown as green squares.

We can test whether the resistive features are a result of changes in pore water salinity, porosity or clay content for a section of the resistivity model in line 2, where ground-truthing information is available. Due to the measurement error associated with the acquired data and the physics that dictate the diffusive nature of electromagnetic signal propagation, an ensemble of best-fit models exist that describe the measured CSEM data within its uncertainty equally well. We validate the resistivity-depth variations of our CSEM inversion model for line 2 with the resistivity measurements from borehole U1353 (Fig. 5). For the upper 100 m, we only have core porosity and salinity information. For this region, we converted the pore water salinity values from borehole U1353 to a resistivity-depth model using Archie’s Law^45^. We used two different porosity models: one based on the porosity–depth function derived from U1353 (ref. ^28^), shown in blue, and the measured porosity values (MAD) for U1353, shown in green (Fig. 5). The resistivity estimates from our inversion model and the resistivity variation from the core data agree quite well above 100 m bsf, and we can resolve the subtle resistivity variations caused by a salinity variation of 10 psu. We can thus infer that resistive feature 2a corresponds with brackish pore water in borehole U1353 (Figs. 2c and 4a). The uppermost section of the inversion model (<20 m) is likely an erroneous structure that appears due to over-fitting of the data or another systematic inversion artefact (Fig. 5).

Below 100 m bsf, we can validate our inversion resistivity model against the resistivity log measured by the IODP induction tool. The induction tool shows comparatively large variations in resistivity (Fig. 5), which is related to the fine layering of sediments with variations in clay content and grain size (Figs. 2a and 3e). The variation in the resistivity log is likely more attributable to variable clay content, since there is a correlation between resistivity and natural gamma radiation^40^ (Fig. 5). Clay tends to reduce resistivity through conductive pathways along the surface of negatively charged clay particles, causing electrical anisotropy in a predominantly vertical direction. Thin, intercalated clay layers may be directly picked up by the induction tool with its short source–receiver offsets, causing the wide scatter in the resistivity log, while the horizontal inline electric field component measured with the seafloor-towed CSEM system is mainly sensitive to vertical current flow in the subsurface. The resistivity model derived from the CSEM data tends towards the higher resistive layers as seen in feature 2b (Figs. 4a and 5).

### Estimation of pore water salinity

**Fig. 6:**
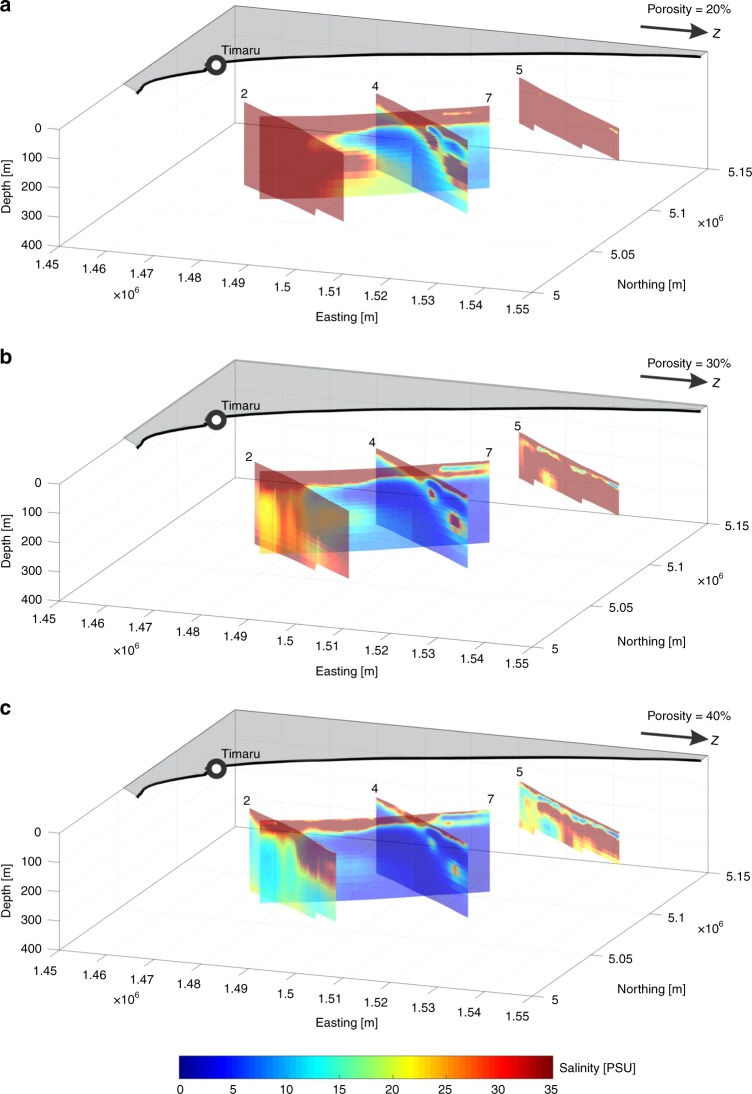
Models of estimated sub-seafloor pore water salinity. Fence diagrams of salinity depth profiles derived from the resistivity models in Fig. 4 by applying Archie’s Law^45^ and the Fofonoff and Millard algorithm^47^. We used constant porosities of **a** 20%, **b** 30% and **c** 40%.

In the absence of borehole data for the resistive feature 2c and along lines 4, 5 and 7, we apply Archie’s Law^45^ and the Fofonoff and Millard algorithm^47^ to calculate pore water salinities from the resistivity models for porosities of 20%, 30% and 40% (see Methods; Fig. 6). We observe fresh to brackish (<10 psu) offshore groundwater in lines 4 and 7 for all porosity scenarios. This result indicates that the high resistivity features along lines 4 and 7 (Fig. 4b, d) are primarily related to low pore water salinities. More brackish (10–22 psu) offshore groundwater is visible in lines 2 and 5, if a porosity of ≥30% is considered (Fig. 6b, c). In view of the predominant sediment facies (silts and fine sands; Fig. 3e–h), we consider the 40% porosity estimate as the most representative (Fig. 6c). We observe shore-parallel variations in pore water salinity along line 7, and between lines 2, 4 and 5. The freshest groundwater always occurs along, and laterally from, line 4. The resistivity models in Fig. 4b, c, and the derived pore water salinity models in Fig. 6c, suggest that two smaller OFG bodies occur above a main OF0G body in lines 4 and 7. They are up to 15 km long, 50 m thick, have lenticular cross-sections and correspond to fine sand bodies (Fig. 3f, h).

### Hydrological models

The goals of the hydrological models are to quantify the hydraulic characteristics of the OFG system and to provide insights into the relative importance and timing of freshwater transport and emplacement. We constructed three shore-normal cross-sectional hydrological models of groundwater flow and solute transport based on lines 2, 4 and 5 (see Methods). The computed hydraulic characteristics are total dissolved solids concentrations, residence times and groundwater velocity (computed by dividing specific discharge by porosity). The governing transport equations, boundary and initial conditions are described in the Methods section.

**Fig. 7:**
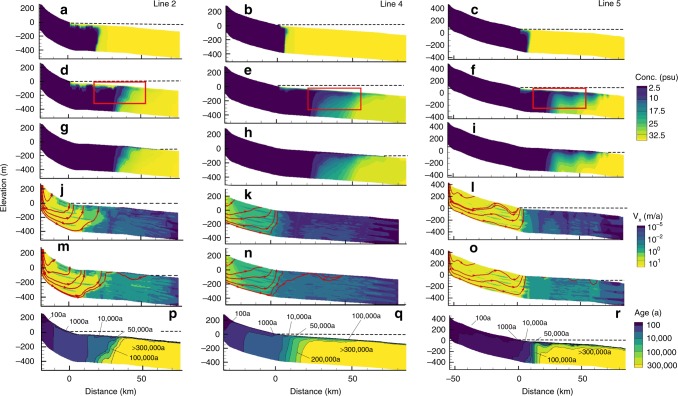
Hydrological model results for lines 2, 4 and 5. **a**–**c** Computed present-day salinity after 1 Ma using fixed, modern sea-level conditions. **d**–**f** Computed present-day salinity after 1 Ma using time-varying sea-level conditions (see Supplementary Fig. 4). The offshore section that corresponds to the CSEM profiles is indicated by a red rectangle. **g**–**i** Computed salinity conditions at the last glacial maximum (21 ka before present). Computed groundwater velocities (*v*) from **j**–**l** transient scenario today and **m**–**o** during the last glacial maximum. The red arrows in **j**–**o** are groundwater streamlines, which are everywhere parallel to groundwater flow directions. While the streamlines appear to converge to a single point near the bottom of the model domain near the coastline, distinctive flow tubes exist that are too thin to be distinguished. The convergence of stream tubes is due to a reduction in flow rates associated with the transition from a high permeability to lower permeability environments. **p**–**r** Computed present-day mean groundwater residence times from the transient simulation. Dashed line denotes sea level.

We first imposed a fixed modern sea-level condition for 1 Ma and refer to this as the steady-state scenario (Table 2). In the steady-state scenario, OFG extends 10–20 km from the coastline and is associated with submarine groundwater discharge due to onshore water table head gradients (Fig. 7a–c). The relatively short distances of observed OFG, in comparison to prior studies^48^, are due to a lateral decrease of permeability offshore (Supplementary Fig. 5b). Flow driven by shore-normal head gradients is dominant. Sequestered offshore fresh (<1 psu) and brackish (<10 psu) water for the steady-state scenario varies between 0.43 and 2.78 km^3^ km^−1^ (volume per km of coastline) (Table 2). Freshwater is mainly sequestered in coarse-grained units close the shoreline.

**Table 2:** Model estimates of the volume of OFG.

Profile	Scenario	*C*_T_ (psu)	*V*_F_ (km^3^ km^−1^)
Line 2	T	1	3.25
Line 2	T	10	5.06
Line 2	SS	1	1.57
Line 2	SS	10	2.78
Line 4	T	1	3.37
Line 4	T	10	4.65
Line 4	SS	1	0.56
Line 4	SS	10	0.43
Line 5	T	1	3.11
Line 5	T	10	4.62
Line 5	SS	1	0.92
Line 5	SS	10	1.23
Average	T	1	3.14
Average	T	10	4.78
Average	SS	1	0.98
Average	SS	10	1.52

*T* transient sea-level model run, *SS* steady-state model run with fixed present-day sea-level boundary condition enforced, *CT* threshold concentration for freshwater volume calculation, *V*_*F*_ volume of freshwater (in km^3^ per km of shoreline) below modern sea-level for a given threshold concentration.

For the transient shore-normal scenarios, where sea level was varied over the past 1 Ma due to Pleistocene climate change^49^ (Supplementary Fig. 4), fresh to brackish water is sequestered in both coarse and fine-grained facies, resulting from diffusive and dispersive processes. This is evident by the lack of fingering between relatively thin coarser and finer units (Fig. 7d–f). Differences in salinity patterns between the three profiles are due to differences in stratigraphy and shore-normal gradients. After 1 Ma of sea level change, the OFG extends up to 60 km from the coastline. Sequestered offshore fresh and brackish water ranged between 3.11 and 5.06 km^3^ km^−^^1^ (Table 2). Most of the OFG is located within 20–40 km from the shoreline. Computed mean groundwater age for the transient simulations indicate that much of the OFG is younger than 300 ka (Fig. 7p–r). Computed groundwater velocities range between 100 m a^−1^ onshore to <10^−5^ m a^−^^1^ offshore at present (Fig. 7j–l). In the uplands, a portion of the topographically driven flow discharges before reaching the ocean and this does not change during sea level lowstands. During sea level lowstands, topographically driven flow takes place across much of the continental shelf, where groundwater velocities are about an order of magnitude higher than during highstands (Fig. 7m–o; Supplementary Fig. 5c). Shallow, local topographically driven flow cells developed in what is today an offshore environment due to local topographic variations on the continental shelf. During sea level highstands, onshore topographically driven flow patterns change slightly (Fig. 7j–l). In the offshore environment, however, reversals in lateral flow directions take place due to reduction in shore-normal flow rates (Fig. 7j–l; Supplementary Fig. 5c) and the presence of lateral density gradients. It is important to point out that this is not haline convection^50^. Calculated grid Rayleigh numbers (see Methods) are sub-critical for fine-grained sand, silt, and clay clastic facies (Supplementary Fig. 6a). Computed horizontal Peclet numbers (see Methods) in the mid-shelf region vary between 10 and 1000 (Supplementary Fig. 6b), which indicates that advective transport dominates. Models of solute transport that rely solely on vertical diffusion and use realistic diffusion coefficients (10^−^^10^ m^2^ s^−^^1^) significantly underpredicted the depth of OFG relative to observed conditions (Fig. 2c; Supplementary Fig. 9; Supplementary Note 1). Modern salinity conditions are not in equilibrium with present-day sea level conditions.

### Comparison of CSEM data and hydrological model results

The model-derived salinity patterns for lines 4 and 5 (Fig. 7e–f) are similar to the estimated pore water salinities for the 40% porosity scenario (Fig. 6c). For line 2, the model-derived salinity field pattern compares favourably with the pore water salinity profiles in boreholes U1353 and U1354 (Fig. 2c). For the upper 100 m of borehole U1353, the CSEM inversion model agrees with the resistivity variation estimated from the core data (Fig. 5), and the estimated pore water salinity (Fig. 6c) compares well with the pore water salinity values (Fig. 2c).

**Fig. 8:**
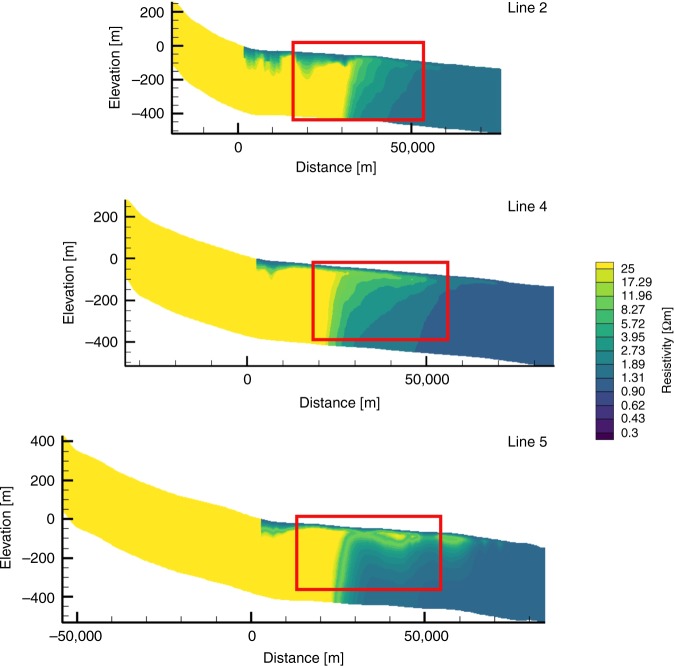
Fluid resistivity profiles computed from the hydrological model results in Fig. 7d–f. Distance refers to the shore-normal direction. The present-day shoreline is located at *x* = 0 km, with negative values of distance referencing the onshore system.

We also converted the salinity profiles derived from the hydrological models (Fig. 7d–f) to bulk formation resistivity profiles (Fig. 8) using Archie’s Law (porosity of 40%) and a thin-plate spline model^51^, and compared them with the CSEM inversion models (Fig. 4). For line 2, the computed formation resistivities are similar to those in the corresponding CSEM model further offshore in the eastern half of the profile, but are higher at depth in the western half (Figs. 4a and 8). In line 4, we observe a thinner main resistivity body and slightly lower resistivities in the computed formation resistivities (Fig. 8) in comparison to the CSEM model (Fig. 4b). The computed formation resistivities for line 5 are higher than those in the CSEM model, but the general distribution compares well (Figs. 4c and 8). Interestingly, the computed model reveals shallow resistivity anomalies, which are also observed in the CSEM model.

A number of factors may explain the discrepancies between the resistivity models derived from hydrological modelling and CSEM data inversion. CSEM has been shown to be a useful geophysical tool to map resistivity changes associated with OFG from local to regional scales. However, the diffusive nature of electromagnetic field propagation favours the interpretation of CSEM data using smooth resistivity contrasts. Thus, small contrasts, like the one resulting from the small salinity anomaly observed in U1353, are difficult to resolve (Fig. 4a). Model resolution also depends on the CSEM measurement configuration. The applied inline electric dipole–dipole system is particularly sensitive to the lateral resistivity distribution down to about 300 m bsf. In turn, the significant resistivity contrasts along lines 4 and 7 are well-resolved by the CSEM data and are interpreted as a clear indication of freshened pore water. As indicated by the seismic reflection data, lithology changes within the spatial scales of the survey area and with depth (Fig. 3e–h). The conversion of seafloor resistivity to pore fluid salinity requires knowledge of the porosity, clay contents and Archie coefficients. This information is only available at borehole U1353 and may change across the survey area. Lithological information derived from seismic reflection data can help to reduce the ambiguity of CSEM interpretation, but do not allow for a direct conversion of the observed resistivity to pore fluid salinity. However, converting the resistivity cross-sections to salinities using averaged porosity estimates derived from borehole data or lithologies identified in seismic data (Fig. 6) provide first-order approximations on the salinity distribution, which are otherwise not available from remote measurements.

An important limitation of our hydrological models is that we used a single permeability value for each facies. In reality, sands, silts and clays can display at least two orders of magnitude variations for a given grain size. Our hydrologic models are also sensitive to the choice of permeability represented (see Methods). Additional uncertainty in our model arises from the hydrostratigraphic framework models (which are based on seismic facies classification and may have missed fine-scale geological variabilities), the degree of connectivity between facies represented in the model^52^, and the choice of initial conditions at 1 Ma (see Methods). The paucity of borehole control precluded the development of 3D hydrological models. Our models are thus conceptual 2D models that lack 3D stratigraphic connectivity, which could account for shore-parallel flow regimes. It is also important to point out that none of the geophysical surveys extend close to the shoreline, where most of the freshwater is predicted to occur in our hydrological models.

### Water column data

Attempts at detecting freshwater seepage across the Canterbury Bight from conductivity–temperature measurements in the water column, water column chemistry, and pore water chemistry from surficial sediment samples (see Methods) were unsuccessful. Measurements made by the conductivity–temperature–depth sensor attached to the CSEM system do show zones with brackish water along lines 2 and 7 (Supplementary Fig. 8). However, in view of the spatial correlation with both coarse and fine sediments at the seafloor (Fig. 3d, h), the small differences between our measurements and water column salinity measurements elsewhere on the shelf^46^, and the poorly constrained physical oceanographic baseline conditions, we are unable to determine whether the low salinity measurements are due to localised diffused freshwater discharge or changes in bottom water salinities due to currents.

## Discussion

The OFG system in the Canterbury Bight consists of one main, and two shallower and smaller, freshened groundwater bodies (Figs. 6–7). The main OFG body extends up to a distance of 60 km perpendicularly from the coast, has a maximum thickness of at least 250 m, and its top reaches a maximum depth of 50 m bsf. The cross-sectional shape of the main OFG body is best described as wedge-shaped, becoming thinner and more saline with increasing distance offshore. Considering porosities of 40%, which are realistic for line 7 (Fig. 3h), the main OFG body extends from the shoreline of Ashburton in the NE to offshore of Timaru in the SW, across an along-shelf distance of 72 km in seawater depths of up to 110 m. The borehole and geophysical data, and the hydrological model results, show that OFG occurs in sedimentary layers that mainly include silt and sand, and occasionally gravel and clay (Figs. 2–7). The minimum and maximum OFG volumes, estimated from the geophysical data for porosities of 20% and 40% (Fig. 6a, c), are 56 and 213 km^3^, respectively. The volume of OFG per km thus ranges between 1.22 and 2.96 km^3^ km^−1^. The model-derived estimates were higher (average of 3.24–4.78 km^3^ km^−^^1^), but these included freshwater sequestered both shoreward and seaward of the CSEM profiles. These volumes compare with 519 km^3^ for the entire onshore aquifer of the Canterbury Plains^53^, and 1.6–1.8 km^3^ km^−1^ offshore of New England^8^, 4.4 km^3^ km^−1^ offshore of New Jersey, 9.9 km^3^ km^−1^ offshore of Florida, 6.3 km^3^ km^−1^ offshore of Suriname, 1.0 km^3^ km^−1^ offshore of Jakarta and 3.1 km^3^ km^−1^ offshore of Gippsland^1^. These published offshore groundwater volumes were based on borehole observations along a profile or numerical model simulations. The seawater depth at which OFG in the Canterbury Bight occurs (up to 110 m) exceeds Pleistocene average sea level (~40 m)^4^. This implies that the onshore hydraulic drive in the Canterbury Basin is unusually high, which we attribute to a high onshore topographic gradient of 0.77% (in comparison to 0.05% for New Jersey^27^, for example).

We infer that the origin of the OFG is predominantly meteoric based on the geochemical characteristics of the pore water in borehole U1353. Concentrations of Ca^2+^ and HCO_3_^−^ tend to be lower and higher, respectively, in coastal groundwater compared to seawater^54,55^. Pore water samples from borehole U1353 can be divided into two distinct groups according to their geochemical characteristics (Supplementary Table 1). Pore water samples from group 2 (depth range of 114.6–318.5 m bsf) have average concentrations of 624 mg l^−^^1^ for Ca^2+^ and 138 mg l^−^^1^ for HCO_3_^−^, indicating seawater composition. Pore water samples from group 1 (depth range of 59.7–75 m bsf) have mean concentrations of 383 mg l^−^^1^ for Ca^2+^ and 320 mg l^−^^1^ for HCO_3_^−^, suggesting freshwater mixing. The ratio of HCO_3_/Cl is a significant indicator of freshwater recharge if it is greater than the seawater ratio (0.0069)^56^. Samples from groups 1 and 2 had HCO_3_/Cl ratios of 0.0124 and 0.0041 (Fig. 2f), respectively, indicating that samples from group 1 were likely emplaced by freshwater recharge. The Na/Cl ratio allows us to define the salinity source in water^57–59^. In our case, the Na/Cl ratio varies from 0.86 to 0.88 with an average value of 0.87 and suggests that the water samples of the study area are highly saline^57^. The scatter plot between Na and Cl shows that the group 1 samples have lower values of Na and Cl when compared to group 2 samples (Fig. 2g), which may be due to mixing with freshwater. If the freshwater was emplaced by mineral hydration, the source would have been deeper and the salinity profile would have been the reverse of the one recorded in borehole U1353. Gas hydrate dissociation is also an unlikely source of the OFG, in view of the fact that the concentrations of methane in borehole U1353 are low (Fig. 2e), and that the gas hydrate stability field does not extend to the continental shelf^60^.

The offshore extension of the onshore gravel sequences facilitates migration of meteoric water from the Canterbury Plains aquifers to the main OFG body. A number of observations suggest that topographically driven flow of groundwater from onshore to offshore is taking place at present. First, the water budget for the Hinds Rangitata Plain shows that groundwater outflow to the ocean can reach up to 65% of all discharge from the coastal aquifer^33^. Second, in the Rakaia–Ashburton Plains, groundwater near the coast has a relatively young age (<50 a), which, combined with the general absence of surface springs, suggests substantial active offshore flow^61^. Third, our model estimates of groundwater age distribution qualitatively agree with these observations (Fig. 7p–r). Relatively young groundwater ages are found in aquifers onshore and near the shoreline (100 a near the surface), whereas older waters are found offshore. The OFG system has a wedge-shaped geometry and exhibits an increase in salinity with distance offshore (Figs. 6, 7), which in other settings has been associated to a connection with onshore aquifers^6,20,27^.

The maximum offshore extent for a present-day OFG in the Canterbury Bight, as estimated in the steady-state scenario (Fig. 7a–c, Table 2), is between one-sixth and one-third the extent of the OFG inferred from our data (Fig. 6). This indicates that recharge from onshore aquifers at present can only account for a small fraction of the OFG. Our transient model results suggest that the majority of the OFG was emplaced during the last three glacial cycles (Fig. 7p–r). During low sea levels, topographically driven shore-normal flow was higher than at present due to an increase in the hydraulic head and steep onshore gradients (Supplementary Fig. 5c). This played a key role in driving freshwater offshore and extending the OFG further out into the continental shelf. Enhanced infiltration, due to a more extensive area exposed to meteoric recharge, played a less important role. Local flow cells developing on the exposed shelf helped to enhance freshwater infiltration. As sea level rose, there was a reduction in the topographically driven flow. Lateral differences in salinity on the shelf drove groundwater laterally and in some cases shoreward (Fig. 7j–l). The smaller OFG bodies in coarse-grained sediments in the shallowest layers (Figs. 3, 4 and 6), on the other hand, may be explained by higher groundwater flow in a direction oblique to the profile via permeable conduits, or incorporation as fresh connate water during deposition during the last glacial cycle.

The difference in along-shelf OFG salinity distribution across the study area, with the freshest offshore groundwater located in the vicinity of line 4 (Figs. 4d and 6), may be explained by three factors:

Sedimentary framework of the Canterbury Bight: The sedimentary and permeability architecture of braided alluvium is inherently heterogeneous^62^. Across the Canterbury Plains, the braided alluvium predominantly comprises medium to coarse sandy gravels^63^, which correspond to tabular primary channel fills that formed by deposition in first-order channels^64^. Ten per cent of the total volume of the braided alluvium consists of narrow and arcuate conduits of coarse gravel (also known as open-framework gravel)^63^, which is well-sorted material from the fastest flowing river channels^65^. These conduits, which can be up to five times more permeable than the sandy gravels, account for the majority of groundwater flow^63^. Floodplain and lacustrine shales, on the other hand, can act as laterally extensive permeability barriers^64^. At the regional scale, conduits provide a strong permeability anisotropy to the aquifer, but they only constitute discrete, identifiable flow channels at the local scale (metres to hundreds of metres). These sedimentary structures are expected to extend offshore^30^. A second source of heterogeneity are high permeability corridors associated to unconfined braided rivers that act as preferential groundwater flow pathways. These buried features, which can be up to 10 km wide and sub-perpendicular to the coastline, were developed during interglacials and infilled with coarse-grained sediments during glacials^31^. In line 7, we interpret the thin and isolated bodies of facies C occurring at multiple depths (Fig. 3d, h) as high permeability corridors^30,40^. Both conduits and high permeability corridors can account for lateral changes in permeability and OFG salinity along the Canterbury Bight, although only the corridors can be detected in our geophysical data.

Recharge from onshore aquifers and rivers: Groundwater flow estimated in the vicinity of the Rangitata River, which is located onshore of line 4, is lower than that for the Ashburton River, found onshore of line 5 (113 vs. 176 m^3^ a^−^^1^, respectively^34^) (Fig. 1). Piezometric contours for a regional groundwater surface show a general flow parallel to the topographic gradient towards the coast, and small deviations in the contours, indicative of lower heads, around the river mouths^66^. These two observations suggest that onshore recharge is not an important contributor to along-shelf variations in OFG salinity at present. However, Rangitata River has a median flow rate of >5 times that of Ashburton, Opihi, Orari, and Hinds Rivers (Fig. 1 (ref. ^67^). Since Rangitata River has the largest catchment area, it likely had the highest flow rate during glacial periods as well^68^. In view of this, and the fact that aquifer systems onshore are primarily replenished by infiltration from rivers^34^, we propose that the Rangitata River had the potential to provide more sustained recharge to the adjacent groundwater than the Ashburton River when these rivers extended across the continental shelf during lower sea levels.

Structural control: The only structural features identifiable in our study area include a series of normal faults, as inferred from the offset seismic reflectors in line 7 (Fig. 3h; Supplementary Fig. 1). These faults extend vertically up to sequence boundary U16, which has an age between 0.44 and 1.05 Ma^37–39^. These faults are either previously unmapped, or comprise extensions of the fault mapped by Lu and Fulthorpe^37^. The faults coincide with the SW limit of the main OFG body (Figs. 4d and 6). They likely acted as a source of shallow groundwater salinisation due to flow of saline water along hydraulically conductive faults from overpressured sediment below^27^. It is also possible that the faults provide a barrier to the lateral flow of groundwater to the SW as a result of displacement and steepening of the permeable strata, and/or clay smearing^52,69^.

Topographically driven flow and solute transport can also result in three-dimensional redistribution of salinity profiles^70^. We note that the Canterbury Plains are characterised by topographic gradients of >0.05% parallel to the coast. We therefore hypothesise that topographically driven flow systems could have developed on the Canterbury Bight during sea level lowstands and may also account for along-shelf variability in OFG salinity.

This study has demonstrated that the integration of time-domain CSEM data with seismic reflection data and hydrologic modelling, constrained by borehole data, is a powerful approach to quantitatively characterise OFG. The geophysical data can determine the 3D geometry, extent and dimensions of the OFG and identify controls of salinity distribution, whereas hydrological modelling can provide insights into the mechanisms and timing of groundwater emplacement. In our case, the method has allowed us to map a previously unknown OFG system offshore of Canterbury, which has the potential to provide a source of freshwater to one of the driest regions in New Zealand in the future. The high-resolution characterisation of this OFG system has revealed a more extensive and fresher OFG body than could previously be inferred from borehole data or analytical modelling alone. Our results also suggest that aquifer structures and OFG characteristics are more complex and variable in comparison to what has previously been documented in other margins. Geological characterisation of the sub-seafloor, particularly in terms of porosity and permeability, is fundamental to OFG system investigation. Both sedimentary structures and faults exhibit spatial heterogeneity along the shelf and play a key role in controlling variability in OFG characteristics. The latter supports inferences based on numerical modelling^71^, and suggests caution in extrapolating the characteristics of OFG systems along continental margins. Modelling of the evolution of the OFG system during successive glacial cycles is crucial to understanding conditions at present. Such efforts would benefit significantly from 3D representation and consideration of the temporal evolution of seafloor geomorphology and stratigraphy. The remarkable depth at which OFG occurs in the Canterbury Bight is a result of high shore-normal topographic and hydraulic gradients. Prior estimates of OFG volumes only included 16% of the present-day coastline and focused on passive continental margins^1^. Including coastlines along active margins with their steep coastal topographies is likely to result in a significant revision of global volumetric estimates of OFG.

## Methods

### Marine data

The following data were acquired during oceanographic expedition TAN1703, which took place on board the R/V Tangaroa between 7 April and 1 May 2017.

Sub-bottom profiles: A Kongsberg Maritime TOPAS PS 18 Parametric sub-bottom profiler, with a linear frequency-modulated chirp (LFM) with a frequency range of 2.0–6.0 kHz and a chirp length of 15/20 ms, was used to acquire the sub-bottom profiles. The data were sample with 40 kHz and the recording length was 300 ms. The TOPAS PS 18 beam was stabilised for heave, roll and pitch movements via motion data fed from the POSMV. In areas with a steep slope gradient, the acoustic beam was steered manually. The data were processed using a matched (wavelet) filter, an automatic digital gain, a time-varying bottom tracked gain and subsequently converted into instantaneous amplitude data. This resulted in a vertical resolution of up to 20 cm in the acquired profiles.

Multichannel seismic reflection profiles: 600 km of high-resolution MCS data were acquired using a mini GI-gun (13/35 cubic inch), deployed at 1.5 m water depth and shooting with a pressure of 1800–2000 PSI (124 to 138 bar). Three 100 m long active solid-state sections of the GeoEel digital seismic streamer (Geometrics), containing eight hydrophone groups per section (spacing 12.5 m), served as receiving unit. The acquisition parameters were set to a shot interval of 3 s and a record length of 1.5 s. Data processing was carried out using GLOBE Claritas^TM^ and included the following operations: conversion from SEGD to SEGY, co-ordinate conversion, definition of streamer geometry, special divergence corrections and band pass filtering (corner frequencies of 50, 100, 500 and 700 Hz), common depth point binning (6.25 m bin size) and sorting, normal move-out correction and stacking, and quality control of processed data. Post-processing included swell correction, deconvolution, migration and AGC/Balancing. The processed seismic profiles have a vertical resolution of 2–2.5 m. In addition to the multichannel seismic reflection profiles collected during TAN1703, we also used the EW00-01 multichannel seismic reflection data set, which was acquired in 2000 by R/V Maurice Ewing across the outer shelf and slope area of the Canterbury Bight^72^. The survey includes 57 profiles (total length of ~3750 km) with a spacing of 0.3 to 0.7 km between the individual lines. For data acquisition, two GI air guns (45/45 in^3^) and a streamer containing 96–120 channels in 12.5 m groups, each containing 26 hydrophones, were deployed. A shot interval of 5 s and a record length of 3 s were set during acquisition. The data processing was carried out with the Focus software resulting in stacked profiles that provide >1.6 s seafloor penetration and a vertical resolution of ~5 m.

CSEM data: 175 km of seafloor were surveyed with a bottom-towed, time-domain, CSEM provided by BGR^73^. GEOMAR’s deep-sea current transmitter was used to generate 20 Ampere and 50% duty cycle square wave current signals, which were injected into the subsurface via a 100 m horizontal electrical dipole^74^. Four receiver units (HYDRA) recorded the inline electrical field responses at predefined offsets of approximately 150, 250, 400 and 650 m from the source centre at a sampling rate of 10 kHz. Stationary measurements were carried out every 500 m along lines 2, 4 and 5, and every 1000 m along line 7. Additional data were recorded in between waypoints while the array was moving, but they were neglected in the interpretation due to inferior signal-to-noise ratios. The CSEM transmitter at the front of the seafloor array was equipped with a Seabird SBE37 conductivity–temperature–depth probe. Data processing was carried out using an in-house software at GEOMAR that synchronises the measured time series with the source signal. The raw data are subsequently robustly processed to final step-off transients at each waypoint and for each receiver. Subsequently, a 2D inversion was conducted for the time-domain CSEM data using an extension of MARE2DEM^75^ to derive a resistivity cross-section up to depths of approximately 350 m.

Conductivity–temperature–depth profiles and Niskin bottle samples: Conductivity–temperature–depth profiles were acquired at 27 stations using a combined a Seabird Electronics Inc. (SBE) 911plus instrument and a 24 × 10 l SBE 32 Carousel water sampler. The sensor configuration consisted of TC-ducted primary temperature and conductivity (SBE 3plus and SBE4, respectively) and a pressure sensor (Digiquartz). Measurements of conductivity, temperature and pH of the waters sampled at these stations were made on board, whereas analysis of anion (Ca^2+^, Na^−^) and cation (Cl^−^, SO_4_^2−^) concentrations was carried out by Hill Laboratories in Hamilton, New Zealand, using inductively coupled plasma mass spectrometry.

Analyses of pore water from sediment cores: Coring was carried out at six sites using NIWA’s in-house, purpose-built piston coring system, which has a 3–6 m long barrel. Pore water was extracted from the recovered sediment using Rhizon samplers. Measurements of conductivity, temperature and pH were made on board, whereas analysis of anion (Ca^2+^, Na^−^) and cation (Cl^−^, SO_4_^2−^) concentrations was carried out by Hill Laboratories in Hamilton, New Zealand, using an inductively coupled plasma mass spectrometry.

Granulometric analyses of IODP 317 samples: 41 samples from depths ranging between 0 and 200 m bsf from borehole U1353 were analysed for grain size distribution using sieves, following the ASTM D0422, and a Malvern Mastersizer 3000.

### Estimation of pore water salinity

We applied Archie’s Law^45^ and the Fofonoff and Millard algorithm^47^ to calculate pore water salinities from the resistivity models. The values for porosity and the constants in Archie’s Law were derived from borehole U1353 (refs. ^28,40^) (Fig. 2). For each interpreted seismic facies, we assigned the following values for porosity: Facies A and B—45% for silts and clays; Facies C—40% for fine sands; Facies D—35% for coarse sands; Facies E—20% for gravel (with sands)^28,63^. These porosity values are on the lower end of marine clastic deposits reported by Spinelli et al.^76^. For each line, we estimated pore water salinity by using three different porosity values, representative of gravels to fine sands: 20% for the lowest porosity, 30% for an intermediate porosity, and 40% for the highest porosity. We also took into consideration the variation of porosity with depth in borehole U1353 (Fig. 2d), which varies according to a porosity–depth function^28^, and assumed that this function applies to all CSEM lines. This calculation gives us the upper and lower bounds of the spatial extent of the OFG system. We adopted this approach for three reasons. First, due to insufficient data quality and offset in the seismic reflection data, it was not possible to derive velocities that could be used to invert for porosity elsewhere. Second, the resolution of CSEM data is not high enough to resolve thin sandy layers in the sub-seafloor, which could cause vertical anisotropic resistivities. Third, horizontal electric dipole-dipole methods, like the one used in this study, are more sensitive to vertical resistivity than horizontal resistivity^77^. As a result, we cannot impose a high-resolution porosity model, inferred from the seismic reflection profiles, on a low-resolution resistivity model.

### Generation of hydrostratigraphic framework models

We generated the seafloor sections of the hydrostratigraphic models (Supplementary Figs. 2 and 3) using multi-attribute seismic facies classification. First, the seismic reflection profiles were sub-divided into five seismic facies based on amplitude characteristics, lateral continuity, reflector geometry and two seismic attributes—instantaneous frequency and envelope^78^. The amplitude characteristics and reflector geometry of the seismic facies are the following: Facies A—parallel, continuous, low-amplitude reflectors; Facies B—parallel, continuous, moderate amplitude reflectors; Facies C—parallel, continuous, high-amplitude reflectors; Facies D—irregular, continuous, high-amplitude reflectors; Facies E—irregular, discontinuous, high-amplitude reflectors, locally associated with velocity pull-ups. Second, a depth–travel time relationship was determined from the sonic logs and used to correlate features in the borehole logs, recorded in the depth domain, with features in the seismic reflection data, recorded in the time domain. A synthetic seismogram was constructed in boreholes U1353 and U1354 from the sonic log and the density curve calculated from the resistivity log using Archie’s relationship^28^. Third, four of the five seismic facies were correlated with sediment grain size in boreholes U1353 and U1354.

The onshore sections of the hydrostratigraphic model were based on published literature. The connection between the onshore and offshore sections, and the extension beyond and below the seismic data (Supplementary Fig. 2) was based on EW00-01 multichannel seismic reflection data and published stratigraphic models^30^.

The finite element mesh comprised 13041 nodes forming 25600 triangular elements. We used a structured finite element grid composed of 160 elemental columns and 80 elemental rows. Each element had a characteristic dimension of about 5 m (Δ*z*) by 750 m (Δ*x*). The onshore portion of the model domain had a topographic slope and length that varied between 0.007 and 0.01 (Δ*z*/Δ*x*), between 19 and 50 km, respectively (Supplementary Fig. 5a). The submarine portion of the shelf varied in length between 75 and 85 km. The hydrostratigraphic properties were interpolated onto the triangular finite elements using an image analysis programme (Supplementary Fig. 3).

### Hydrological modelling

This section presents the governing transport equations, boundary and initial conditions used in the hydrological models. The model runs considered sea level variations of 120 m over a 1 Ma period (Supplementary Fig. 4, referred to as transient model runs) as well as steady-state models in which sea level was fixed at present-day levels.

Mathematical model: We solved the following freshwater head based, variable-density groundwater flow equation:

1 $${\mathrm{S}}_{\mathrm{s}}\frac{{\partial h}}{{\partial t}} = \nabla \cdot \left[ {\frac{{{\mathbf{k}}\rho _og}}{\mu }\nabla \left[ {h + z\rho _{\mathrm{r}}} \right]} \right],$$

where *h* is the equivalent freshwater hydraulic head, S_s_ the specific storage, **k** the permeability tensor, *μ* the fluid viscosity, *z* elevation, *ρ*_r_ relative fluid density (*ρ*_r_ = [(*ρ* − *ρ*_o_)/*ρ*_o_)], *ρ*_o_ is the density of groundwater at standard state conditions (0 °C, 0 mg l^−^^1^, 10^5^ Pa), and *ρ* is the fluid density. The Darcy flux vector $(\vec q)$ depends on both head and relative fluid density gradients:

2 $${\mathbf{q}} = - \frac{{{\mathbf{k}}\rho _{\mathrm{o}}g}}{\mu }\nabla \left[ {h + \rho _{\mathrm{r}}z} \right].$$

We solved the following solute transport equation:

3 $$\phi \frac{{\partial C}}{{\partial t}} = \nabla \cdot \left[ {\phi {\mathbf{D}}\nabla \left[ C \right]} \right] - \vec q\nabla {\mathrm{C}},$$

where *C* is the concentration, **D** the diffusion/dispersion tensor, and *ϕ* the porosity. Note that groundwater velocity is equal to **v** = **q**/*ϕ*. The diffusion/dispersion tensor is given by

4a $$D_{x{{x}}} = \alpha _L\frac{{v_x^2}}{{\bar v}} + \alpha _T\frac{{v_{\mathrm{z}}^2}}{{\bar v}} + D_{\mathrm{d}},$$

4b $$D_{zz} = \alpha _T\frac{{v_x^2}}{{\bar v}} + \alpha _L\frac{{v_{{z}}^2}}{{\bar v}} + D_d,$$

4c $$D_{xz} = {{D}}_{{{zx}}} = \left[ {\alpha _L - \alpha _T} \right]\frac{{v_xv_z}}{{\bar v}},$$

4d $$\bar v = \sqrt {v_z^2 + v_x^2},$$

where *D*_d_ is the molecular diffusion coefficient and *α*_*L*_ and *α*_*T*_ are the longitudinal dispersivities, respectively.

The mean residence time equation solved in this model is given by

5 $$\phi \frac{{\partial A}}{{\partial t}} = \nabla \cdot \left[ {\phi {\mathbf{D}}\nabla \left[ {CA} \right]} \right] - {\boldsymbol{q}}\nabla {\mathrm{A}} + \phi,$$

where *A* is the calculated mean groundwater age^79^.

*RIFT2D* solves the above system of equations using the finite element method. Triangular elements using linear shape functions were employed^80^. The resulting system of algebraic equations is solved directly using Gaussian elimination. Verification of the accuracy of the variable-density flow and solute transport aspects of the code are presented in the appendix of Person et al.^7^.

We also computed average Rayleigh (Ra) and Peclet (Pe) numbers along all three profiles (Supplementary Fig. 6). The Rayleigh and Peclet numbers^55^ are given by

6a $$Ra = \frac{{{\mathrm{\Delta }}\rho g\bar k_zH}}{{\mu D_{\mathrm{d}}}},$$

6b $$Pe = \frac{{\bar v_xL}}{{D_{\mathrm{d}}}},$$

where Δ*ρ* is the density difference between seawater and freshwater, $\bar k_z$ is the vertically averaged permeability per column, $\bar v_x$ is the vertically average velocity, *H* is the average thickness of the profile, *D*_d_ is solute diffusivity, *L* is the cell length, *g* is gravity, and *μ* is fluid viscosity. The Rayleigh number used the average vertical permeability and assumed a sediment column thickness (*H*) of 300 m. The Peclet number used a lateral cell dimension (*L*) of 750 m. Both Rayleigh and Peclet numbers assume a sediment diffusivity of 10^−^^10^ m^2^ s^−^^1^.

Initial and boundary conditions: For groundwater flow, we imposed a specified head boundary condition along the top surface of the model domain. For nodes below sea level, we set the initial heads to be equal to the sea level elevation. For nodes above sea level, we imposed a specified head along the top boundary assuming that the water table topography represents a subdued replica of the land surface^81^. In some preliminary models (not shown), we imposed a recharge boundary condition for nodes above sea level. Using modern estimates of recharge^80^ resulted in computed heads above the land surface at some locations in some simulations. Given the uncertainty of palaeo-recharge during the Pleistocene, we decided that a specified head boundary was more likely to produce realistic flow rates. For the shore-normal cross-sections, we imposed no flux boundary conditions along the base and sides of the model domain. During the past 2.6 Ma, sea level was, on average, 40 m lower than present. During the last glacial maximum (21 ka before present), sea level reached 120 m below modern levels^49^, exposing continental shelf strata.

For solute transport and groundwater residence times, we assigned a specified concentration/age at the top boundary and no flux boundary conditions on all other sides for the shore-normal cross-sections. We assigned an initial concentration of 0 psu for nodes in columns above sea level and 35 psu for nodes in columns that were below sea level. We used the modern sea level elevation of 0 m to assign these initial salinity conditions. While we could have assumed that, at the initial conditions, freshwater occupied sediments to a seawater depth of 40 m, we felt that setting it at the modern shoreline was more conservative and ensured that simulated modern OFG was not an artefact of the initial conditions. We assigned a specified value boundary condition for the groundwater residence time equation of 0 years along the top boundary. We assigned an initial age of 0 years for all nodes. We recognise this is an idealisation and that we have neglected the mean seawater residence time. Because of these imposed initial conditions, actual groundwater age could be higher.

Observed recharge, based on rainfall, evapotranspiration and soil moisture data, vary between 100 and 500 mm a^−1^ across the Canterbury Plains^82^. We assumed a constant temperature of 10 °C. Given the thin model domain (~300 m), temperature increases with depth (~1 °C/300 m) or due to differences between marine and continental environments (~6 °C) would have a small effect on computed fluid density when compared to density differences between seawater and freshwater (~25 kg m^−^^3^). Longitudinal and transverse dispersivity was set to 100 and 10 m for all units, consistent with basin scale models^83^. We assumed a solute diffusivity of 10^−10^ m^2^ s^−^^1^. Specific storage was equal to 10^−6^ m^−1^ for all layers. Our model did not include a sediment loading term that could produce overpressures in fine-grained sediments.

Model parameters: The topographic/bathymetric profiles of the three shore-normal lines and the vertically averaged permeability are presented in Supplementary Fig. 5a, b. The permeability in the horizontal direction varied between 10^−12^ and 10^−16^ m^2^ between gravel and fine-grained clay facies (Table 3). The vertically averaged permeability decreased seaward by about 2.5 orders of magnitude between onshore and the continental shelf (Supplementary Fig. 5b). These permeabilities are on the middle to upper end of the range of permeability measurements made for coastal plain and marine sediments^22,76,84^. Measured permeability anisotropy (*k*_*x*_/*k*_*z*_) from sediment cores is typically around 3–10 for clastic materials^85^. When numerical models lump multiple clastic layers of sands, silts, and clays into a single hydrostratigraphic layer, the anisotropy can go up to 1000–10,000^49^. In our study, however, all individual lithologies derived from the seismic data were represented as individual metre-scale units. We assigned an anisotropy of 80 (*k*_*x*_/*k*_*z*_), which we consider reasonable for metre-scale clastic deposits^86^. The porosity values used in the models are shown in Table 3.

**Table 3:** Material properties assigned to different formations in hydrological models.

Formation name	*k*_*x*_ (m^2^)	Porosity
Clay	10^−^^16^	0.45
Silt	10^−^^14^	0.45
Fine sand	10^−13.5^	0.4
Coarse sand	10^−13^	0.35
Gravel	10^−^^12^	0.2

The contrast between the coarse and fine-grained facies could be as high as 8 orders of magnitude. Had we used a larger range (10^−11^ m^2^ ≤ *k*_*x*_ ≤ 10^−18^ (ref. ^2^; Supplementary Table 2) and assigned a higher anisotropy (1000), OFG in the model would have extended further offshore and the pore water salinities for the site of borehole U1353 would have been too low (Supplementary Fig. 7).

The permeability of clay formation could be as low as 10^−21^ m^2^ (ref. ^87^). However, reducing the value of clay permeability below 10^−16^ m^2^ had a second-order effect on advective transport. In addition, the clay facies are mainly found in the deep offshore environment, where seawater depths exceed 100 m below modern sea level conditions.

We ran simulations with permeabilities that were two orders of magnitude lower than listed in Table 3. These produced diffusion-dominated offshore profiles with a lens of brackish water tapering seaward underlain by seawater. This was not consistent with any of the geophysical observations, which indicate freshwater overlain by seawater.

We also explored higher permeabilities for coarse-grained facies (e.g. gravel *k*_*x*_ = 10^−^^11^ m^2^); however, using an onshore specified head boundary condition consistent with modern water table elevations produced unrealistically high recharge rates (up to about 30 m a^−^^1^), which exceeded precipitation measurements.

## Supplementary information

Supplementary Information

## Data Availability

Most data generated or analysed during this study are included in this published article. The conductivity–depth–temperature profiles, and geochemical data for Niskin bottle samples and pore water from sediment cores, are available from the corresponding author on reasonable request.

## Code availability

Access to the RIFT2D code is available from Prof. Mark Person (mark.person@nmt.edu) upon reasonable request.
