# Supplementary material for: 3D characterisation and quantification of an offshore freshened groundwater system in the Canterbury Bight
Source: Nat Commun. 2020 Mar 13;11:1372. doi: 10.1038/s41467-020-14770-7 (PMC7069953; doi:10.1038/s41467-020-14770-7)
Supplement: Supplementary file 1 — Supplementary Information [file 41467_2020_14770_MOESM1_ESM.pdf]

Supplementary information for:

3D characterisation and quantification of an offshore freshened groundwater system in the Canterbury Bight

by Micallef et al.

Supplementary Tables

Supplementary Table 1 Chemical element concentration of pore water samples from borehole U1353

Group 1								
**Depth (m)**	**pH**	**Cl^-**	**HCO_3^-**	**SO_4^{2-}**	**Ca^{2+}**	**Mg^{2+}**	**Na^+**	**K^+**
59.7	7.3	14401	374	1697	366	863	8104	184
61.2	7.6	14525	358	1745	363	867	8215	195
63.4	7.6	15040	339	1826	376	898	8474	205
66.4	7.6	14902	274	1853	368	885	8431	202
69	7.5	15856	328	1990	420	935	8937	202
72	7.6	15502	248	1932	401	903	8804	208
**Mean**	7.5	15038	320	1840	383	892	8494	199
**Standard deviation**	0.11	511.84	44.88	100.67	21.03	24.22	296.27	7.91
**Group 2**								
**Depth (m)**	**pH**	**Cl^-**	**HCO_3^-**	**SO_4^{2-}**	**Ca^{2+}**	**Mg^{2+}**	**Na^+**	**K^+**
114.6	7.7	18135	141	2305	544	1031	10245	197
134.6	8.0	18558	119	2309	568	1027	10459	187
152.1	7.7	19427	143	2387	610	1056	10951	178
178.4	7.2	19318	140	2345	630	1052	10887	174
189.7	7.4	19214	149	2278	624	1045	10738	182
192.6	7.3	19271	147	2291	628	1051	10800	179
197.8	7.6	19558	151	2330	633	1068	10956	184
219.0	7.5	19610	132	2346	637	1068	10996	176
252.6	7.7	20403	131	2415	670	1093	11478	167
289.9	7.5	20244	129	2364	662	1090	11369	201
318.5	7.6	19753	142	2343	659	1070	11078	209
**Mean**	7.6	19408	138	2338	624	1059	10905	185
**Standard deviation**	0.21	623.71	9.29	39.25	37.70	20.28	338.49	12.06
**P value**	0.7613	<0.0001	<0.0001	<0.0001	<0.0001	<0.0001	<0.0001	0.0257

Concentrations in mg l^{-1} (except for pH). Estimated values of mean, standard deviation and P value (unpaired T-test) are included. Pore water samples between 72 and 114.6 m were not retrieved during IODP Expedition 317.

Supplementary Table 2 Permeability and anisotropy comparison for the transient hydrological model results of line 2

Material	Log(k_x) - Model	Log(k_x) - Test	Anisotropy - Model	Anisotropy - Test
gravel	-12	-11.2	80	1000
coarse sand	-13	-11.4	80	1000
fine sand	-13.5	-12	80	1000
silt	-14	-13	80	1000
clay	-16	-18	80	1000

Supplementary Figures

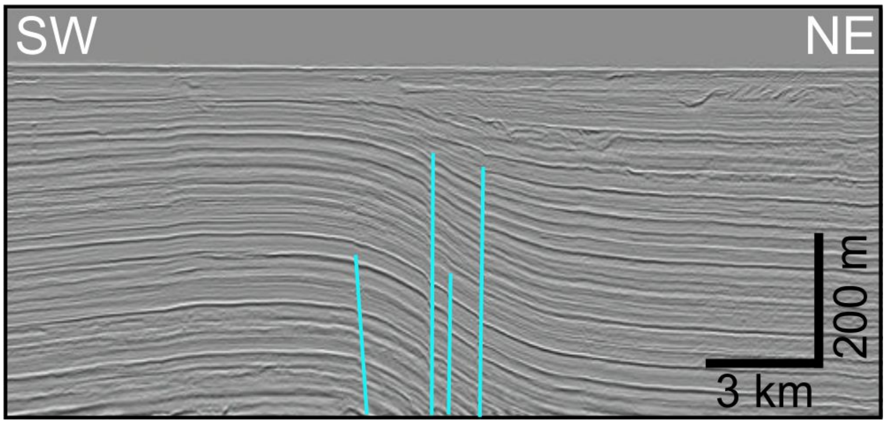

Supplementary Figure 1 Offsets in seismic reflection data. Zoomed section from multichannel seismic reflection profile for line 7, shown in greyscale. Offsets in seismic reflectors are marked by light blue lines. Location in Fig. 3d.

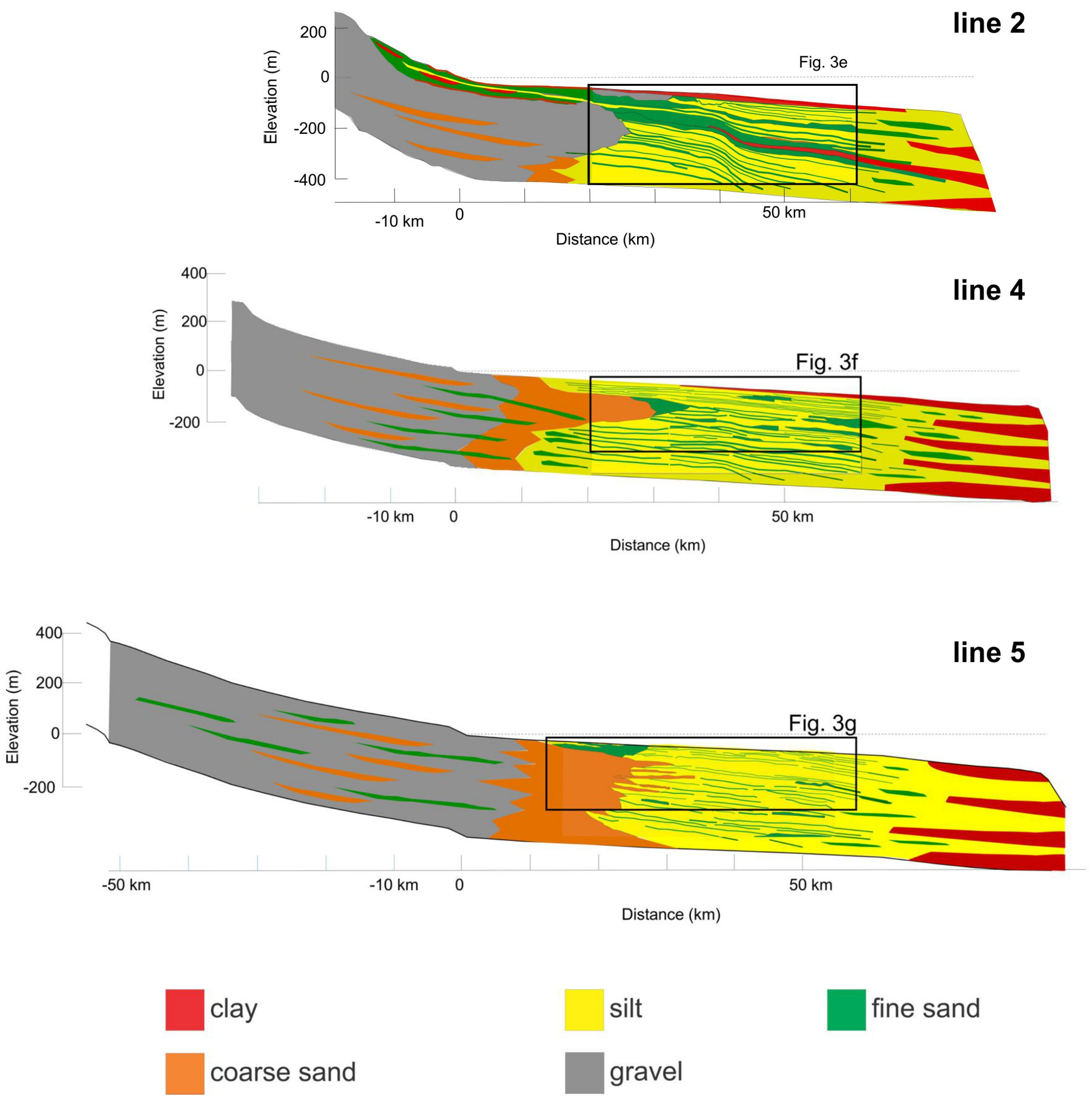

Supplementary Figure 2 Onshore-offshore hydrostratigraphic models for lines 2, 4 and 5. The offshore sections that correspond to the interpreted seismic reflection profiles are indicated by black rectangles.

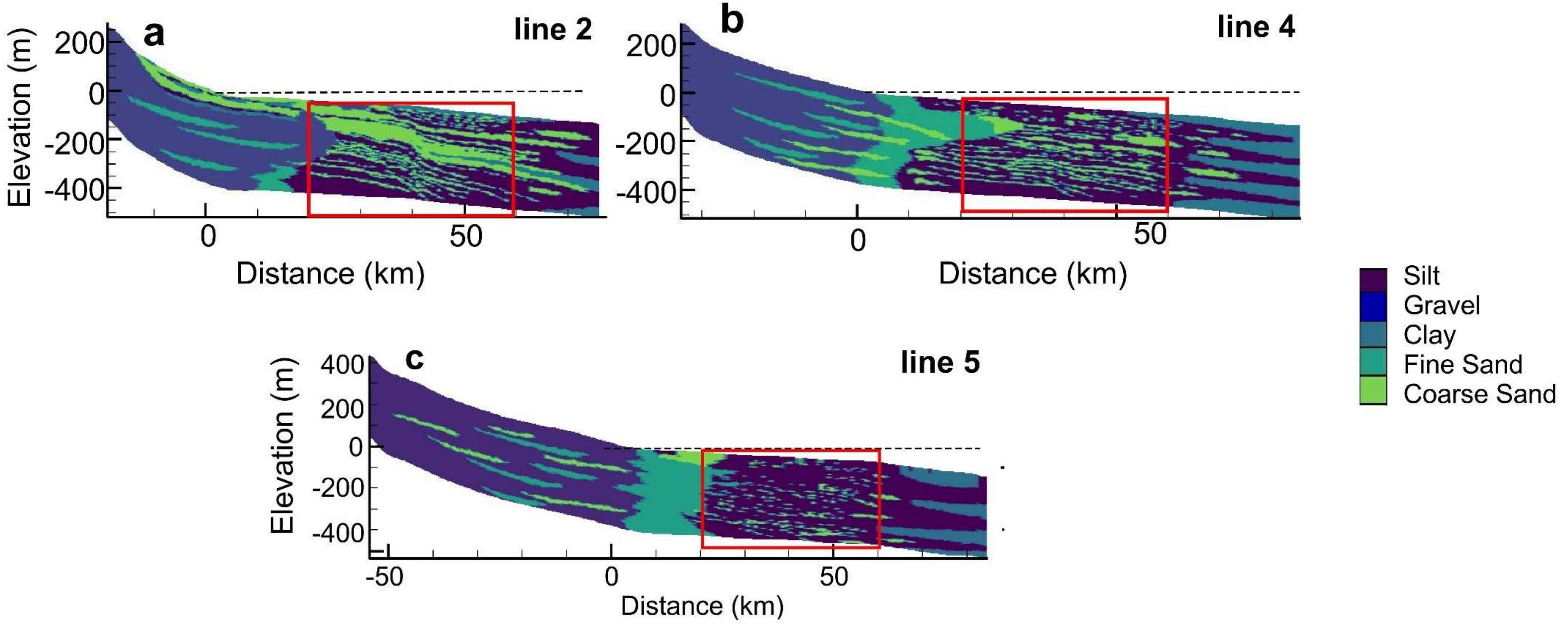

Supplementary Figure 3 Onshore-offshore hydrostratigraphic models for lines 2, 4 and 5 used in hydrological modelling. The offshore sections that correspond to the interpreted seismic reflection profiles are indicated by red rectangles.

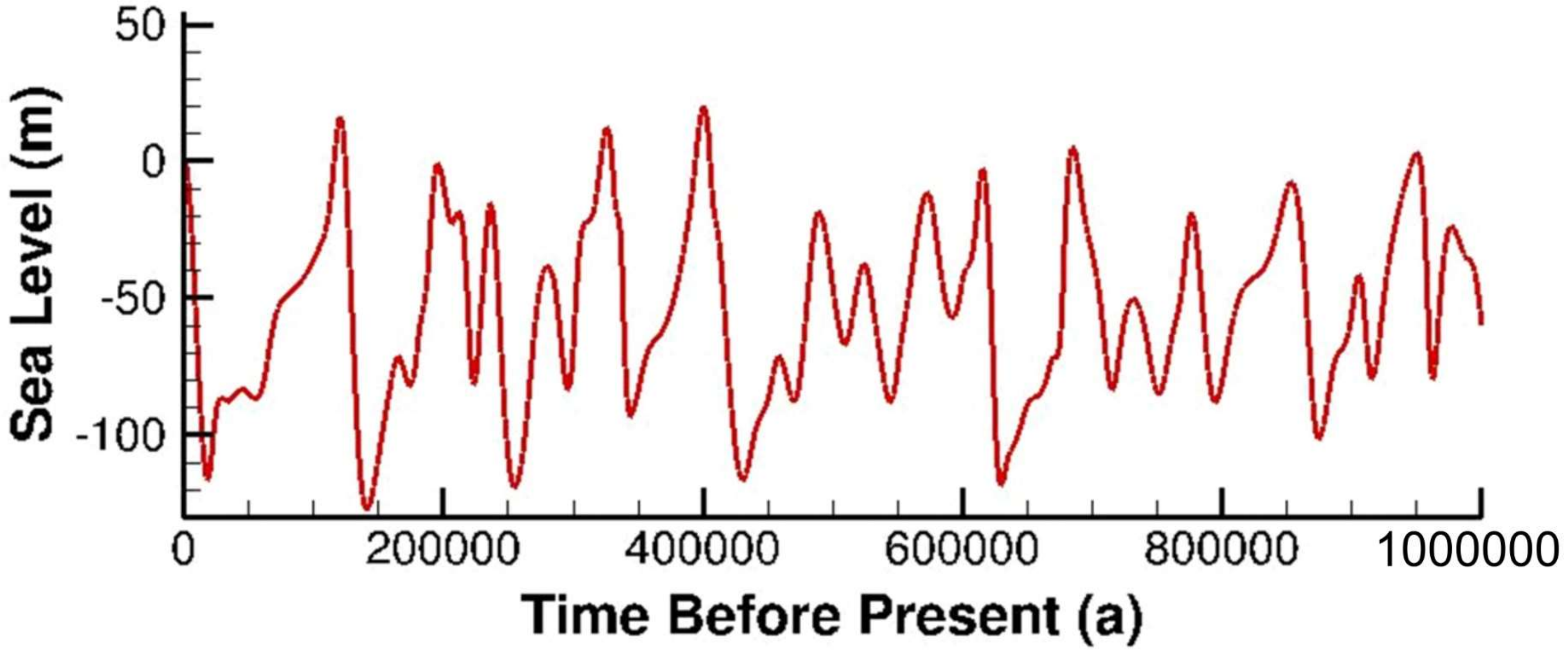

Supplementary Figure 4. Sea level variations during past 1 Ma [49], used in the hydrological models.

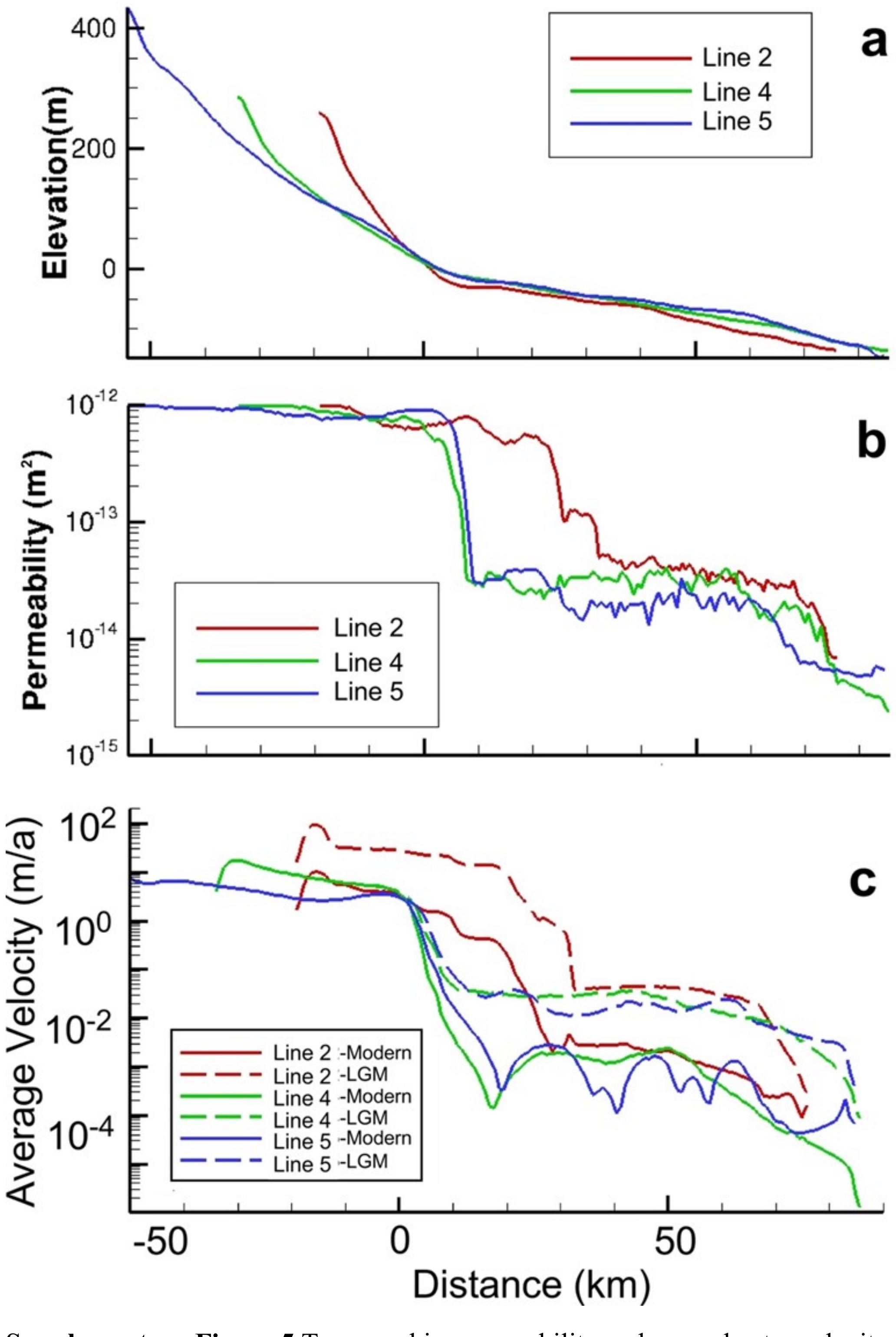

Supplementary Figure 5 Topographic, permeability and groundwater velocity profiles. (a) Topographic/bathymetric profiles and (b) vertically averaged horizontal permeability profiles of model cross sections. Distance refers to the shore-normal direction for lines 2, 4 and 5. The present-day shoreline is located at x = 0 km, with negative values of distance referencing the onshore system. (c) Comparison of shore-normal average groundwater velocity during last glacial maximum. LGM = Last Glacial Maximum.

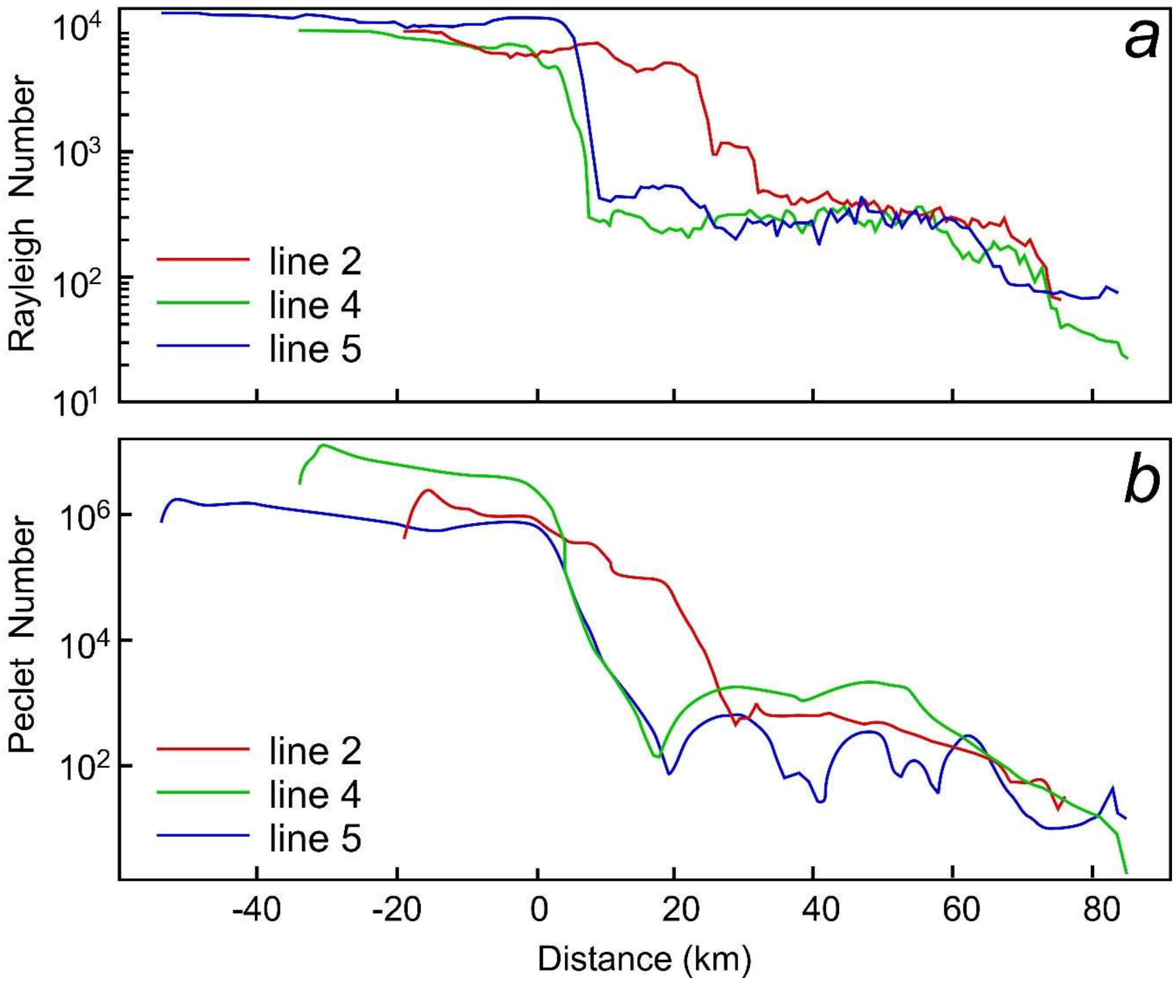

Supplementary Figure 6. Average (a) Rayleigh and (b) Peclet numbers for shore-normal hydrological model simulations. Distance refers to the shore-normal direction for lines 2, 4 and 5. The present-day shoreline is located at x = 0 km, with negative values of distance referencing the onshore system.

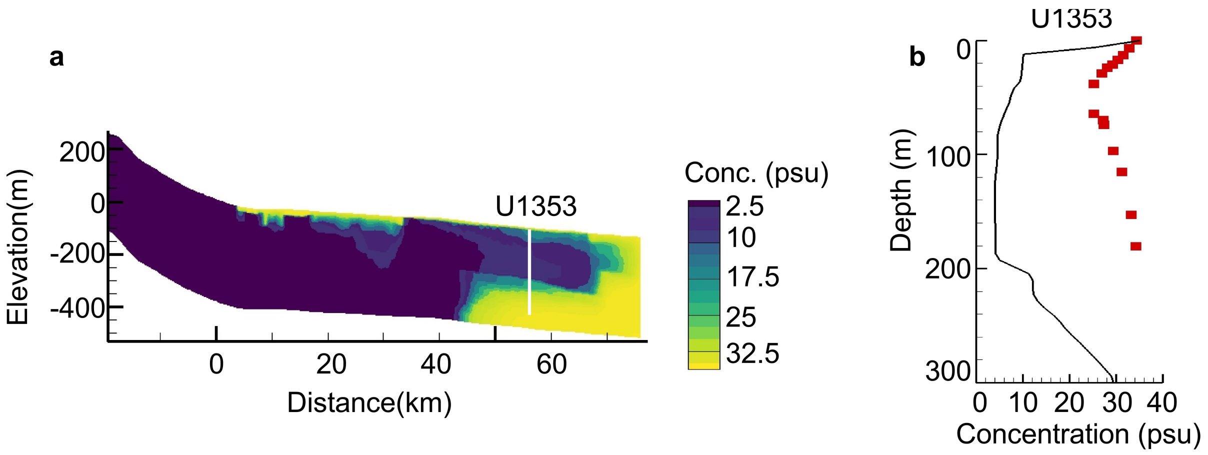

Supplementary Figure 7 Computed salinity from transient model of line 2. (a) Simulated present day salinity distribution for line 2 computed after 1 Ma using time-varying sea level conditions, a permeability range of 10^{-11} $m^2 \leq k_x \leq 10^{-18}$ m^2 and an anisotropy of 1000. (b) Comparison of computed salinity profile (black solid line) and pore water salinity data (red squares) at borehole U1353.

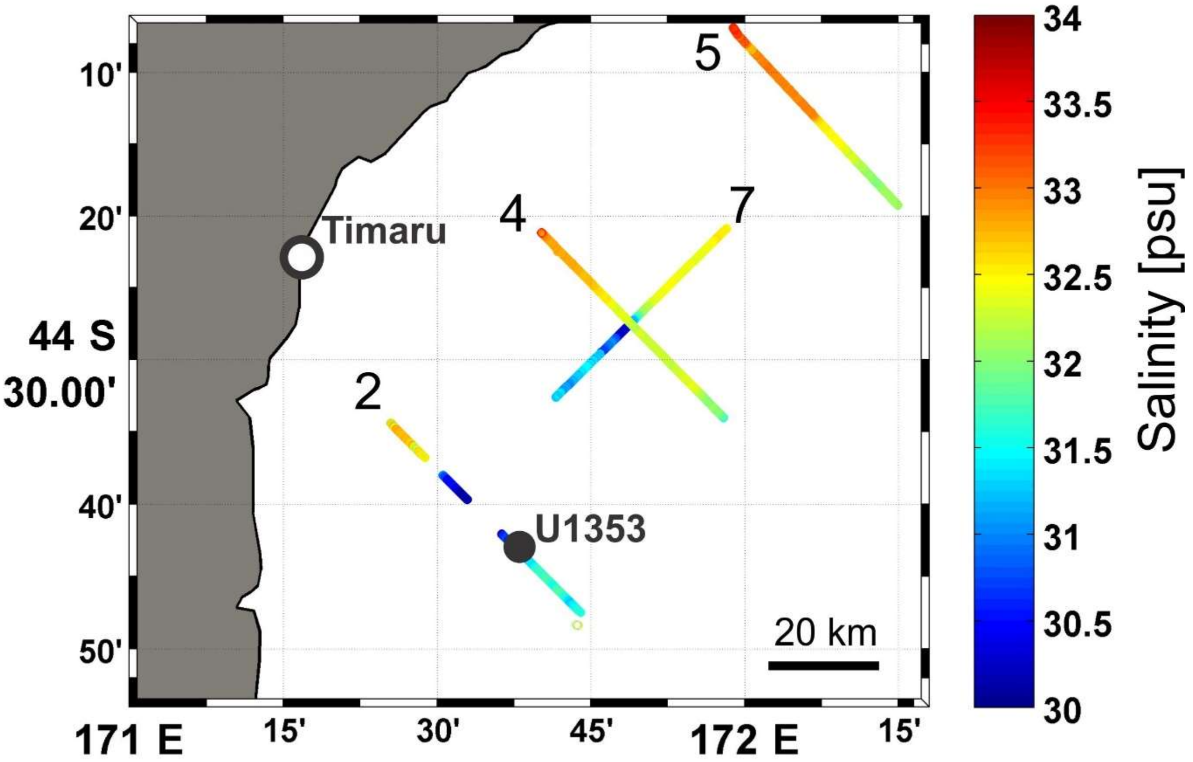

Supplementary Figure 8 Seawater salinity along CSEM lines. Seawater column salinity data derived at the seafloor with the conductivity-temperature-depth sensor on the CSEM system along lines 2, 4, 5, and 7. Location of borehole U1353 is included.

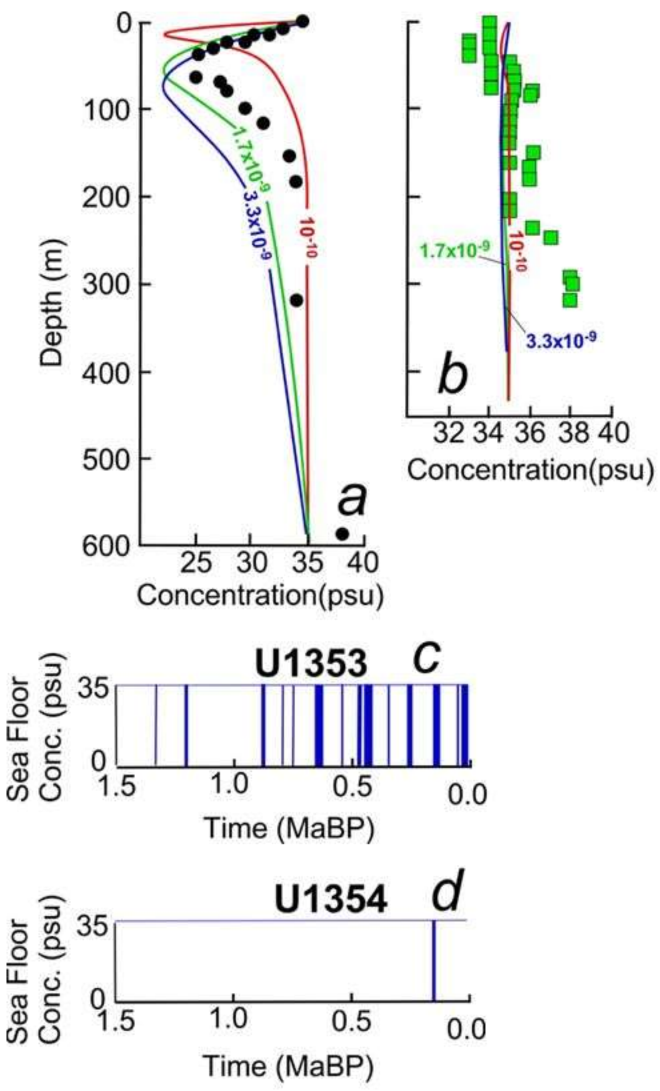

Supplementary Figure 9 Summary of one-dimensional sensitivity study of simulated changes in salinity due to solute diffusion for wells (a) U1353 and (b) U1354, assuming solute transport driven solely by vertical diffusion. A time-varying upper concentration boundary condition of 35 psu was imposed at the sediment-water interface if sea level was above the local land surface elevation at the location of the well. The elevations for wells U1353 and U1354 today are -85 m and -122 m, respectively. Figures (c) and (d) present changes in salinity assigned to wells along the top boundary for U1353 and U1354, respectively. The thickness of the blue lines denotes longer durations of freshwater conditions assigned along the top boundary.

Supplementary Notes

Supplementary Note 1: One-dimensional diffusion models

On first inspection, the salinity patterns within the IODP boreholes appear to be diffusional in nature, with the near shore well U1353 (Supplementary Figure 9a) showing the most freshening in the upper portion of the well and progressively less freshening further offshore in well U1354 (Supplementary Figure 9b). We assessed whether or not these salinity profiles could be explained solely by vertical diffusion processes with a time varying upper salinity boundary condition. We assumed that salinity conditions at the seafloor were a function of Pleistocene sea level fluctuations. Supplementary Figure 9c and S9d present the timing of imposed fresh (0 psu) and seawater (35 psu) conditions at the top boundary of the models. Each model extended down to 600 m below the sea floor. A constant salinity of 35 psu was assigned as an initial condition and at the base of the model domain as a lower boundary condition. The model was run for 1.5 Ma using sea level fluctuations imposed from [49]. We varied sediment diffusivity between 1×10^{-10} and 3.3×10^{-9} m^2/s. We found that an effective diffusion coefficient between about 2×10^{-9} and 3×10^{-9} m^2/s best fit the salinity profile in well U1353 (Supplementary Figure 9a). These diffusion coefficients are as high or higher than free water diffusion coefficients. This suggests that the effects of transverse solute dispersion due to flowing groundwater or advective transport of freshened seawater must also influence the salinity profile in well U1353. Indeed, vertical diffusion cannot explain the salinity profiles in well U1354, which was drilled in a water depth of 122 m.
